# Rational Structure-Based Rescaffolding Approach to *De Novo* Design of Interleukin 10 (IL-10) Receptor-1 Mimetics

**DOI:** 10.1371/journal.pone.0154046

**Published:** 2016-04-28

**Authors:** Gloria Ruiz-Gómez, John C. Hawkins, Jenny Philipp, Georg Künze, Robert Wodtke, Reik Löser, Karim Fahmy, M. Teresa Pisabarro

**Affiliations:** 1 Structural Bioinformatics, BIOTEC TU Dresden, Tatzberg, Dresden, Germany; 2 Helmholtz-Zentrum Dresden Rossendorf, Institute of Resource Ecology, Dresden, Germany; 3 Institute of Medical Physics and Biophysics, University of Leipzig, Leipzig, Germany; 4 Helmholtz-Zentrum Dresden Rossendorf, Institute of Radiopharmaceutical Cancer Research, Dresden, Germany; Indian Institute of Science, INDIA

## Abstract

Tackling protein interfaces with small molecules capable of modulating protein-protein interactions remains a challenge in structure-based ligand design. Particularly arduous are cases in which the epitopes involved in molecular recognition have a non-structured and discontinuous nature. Here, the basic strategy of translating continuous binding epitopes into mimetic scaffolds cannot be applied, and other innovative approaches are therefore required. We present a structure-based rational approach involving the use of a regular expression syntax inspired in the well established PROSITE to define minimal descriptors of geometric and functional constraints signifying relevant functionalities for recognition in protein interfaces of non-continuous and unstructured nature. These descriptors feed a search engine that explores the currently available three-dimensional chemical space of the Protein Data Bank (PDB) in order to identify in a straightforward manner regular architectures containing the desired functionalities, which could be used as templates to guide the rational design of small natural-like scaffolds mimicking the targeted recognition site. The application of this rescaffolding strategy to the discovery of natural scaffolds incorporating a selection of functionalities of interleukin-10 receptor-1 (IL-10R1), which are relevant for its interaction with interleukin-10 (IL-10) has resulted in the *de novo* design of a new class of potent IL-10 peptidomimetic ligands.

## Introduction

Protein-protein interactions (PPIs) mediate most biological processes and, therefore, represent relevant avenues as targets for the development of therapeutics. In order to target large protein-protein interfaces with small molecules in a rational fashion, the most relevant molecular interactions in the functional ligand-receptor complex need to be identified and mimicked appropriately. The rational design of molecules that disrupt PPIs is particularly challenging due to the large and structurally complex nature of the interfaces involved. A decisive strategy has been the identification of key binding residues, which contribute to the majority of stabilizing interactions [1–6]. In some cases, the most contributing residues at the binding interface appear organized in a continuous and well-defined manner in regular architectures (*i*.*e*. secondary structures: α-helices, β-strands and turns). In these cases, the binding epitope containing such key binding residues can be directly translated into a chemical scaffold consisting of the same structural features (for instance, mimetics of protein α-helices, β-hairpins or turns) [7–11]. However, in most cases, the large size, the discontinuous nature, and even the lack of well-defined secondary structure of protein-protein interfaces [12–14] do not allow a straightforward transfer of such relevant residues directly into a small scaffold with high affinity to the targeted recognition site [2, 4]. Antibody-based strategies have been widely exploited for interfering large and structurally complex protein interfaces [15, 16]. Nevertheless, antibodies are suboptimal as drugs mainly due to their low oral bioavailability and cell permeability, slow pharmacokinetics and, sometimes, insufficient stability. From this perspective, small molecules, which can be engineered to target every potential protein of interest, are advantageous over biomacromolecules. In this regard, natural-like small scaffolds or peptidomimetics bear the potential of combining the benefits of both approaches: favorable pharmacokinetic properties of small molecules and the convenient tailoring of biomacromolecules to disrupt protein-protein interactions. Also extensively used to mimic discontinuous recognition epitopes in proteins are phage display techniques, which provide with linear peptides and simple cyclic peptides to target protein-protein interfaces [17–19]. Moreover, small chemical scaffolds such as triazacyclophane have been used to covalently attach discontinuous binding epitopes without further spatial arrangement considerations [20, 21].

A plausible strategy to successfully transfer a large protein recognition site of non-structured and discontinuous nature into a small scaffold in a straightforward manner consists on first identifying a minimum set of functionalities relevant for the molecular recognition, secondly, having in hand a small scaffold able to present the desired functionalities in a suitable three-dimensional (3D) arrangement and to accommodate all chemical features necessary for stability and good complementarity to the targeted site so that it can achieve potent binding affinity. Bringing all these components together constitutes a quite arduous path, and, in order to ease this process, the speedy selection of a suitable scaffold is definitively a determinant step. Once such *ideal scaffold* is in place, a right design rationale strategy is crucial to bringing it into the final desired molecule effectively mimicking the targeted recognition site.

Naturally occurring scaffolds such as β-hairpins, α-helices and structured turns of known protein structures offer a plethora of combinations of chemical functionalities disposed in a particular manner in 3D space and, therefore, they represent a great source of such ideal molecular templates. Thus, protein structure repositories such as the Protein Data Bank (PDB) can be used as a source of *seeding templates* to ease *de novo* design strategies for tackling challenging protein binding epitopes.

Here, we report a minimalist computational structured-based approach to ease the *de novo* design of small scaffolds mimicking challenging protein recognition epitopes of large, non-structured and discontinuous nature. Our methodology is based on regular expressions signifying the three-dimensional disposition of functionalities of a small set of residues (not continuous in the protein sequence) relevant for molecular recognition in a targeted binding site, and it is inspired on the commonly used PROSITE pattern syntax [22, 23]. Our regular expressions are used to query the PDB for matches to small regular architectures in known proteins. Those architectures able to accommodate the desired functionalities in a suitable 3D arrangement are considered as candidate *seeding templates* to lead a rational structure-based rescaffolding design strategy to develop molecules that can effectively mimic the targeted recognition site.

We have applied this innovative and simple rescaffolding approach to *de novo* design of a new class of potent interleukin 10 (IL-10) ligands that mimic the high affinity receptor IL-10R1. This protein-receptor system constitutes a clear example of a challenging molecular interface in terms of receptor mimicry. IL-10 is a pleiotropic cytokine that plays a crucial role in modulation of immune response and pathological inflammatory processes [24–26]. Structurally, IL-10 consists of a symmetric homodimer of two alpha-helical domains [27], and its biological function is modulated by interactions with two cell surface receptors: IL-10R1 (high binding affinity) and IL-10R2 (low binding affinity). The high-resolution 3D crystal structure of IL-10 in complex with IL-10R1 exhibits a quite large binding interface (*ca*. 800 Å^2^) in which the receptor side comprises several discontinuous patches of unstructured nature [28]. To our knowledge, the defiant endeavor of designing small regular architectures as IL-10R1 mimetics has not been previously accomplished. Our work represents a successful example on how to ease the path from discontinuous unstructured protein regions to small regular natural-like architectures in *de novo* rational design of protein mimetics.

## Results and Discussion

### Downsizing the binding epitope to a minimal functional descriptor in 3D

The recognition of IL10-R1 by IL-10 involves 23 receptor residues (S1A and S1B Fig) [28]. The particularly discontinuous arrangement of these residues in sequence, and their quite scattered disposition in 3D space along *ca*. 34 Å, makes it quite challenging to assemble all their functionalities within a small molecule able to mimic the IL-10R1 interaction to IL-10. Based on available structural data [28], residues IL-10R1_Y43_, IL-10R1_R76_, IL-10R1_R96_, IL-10R1_S190_ and IL-10R1_R191_ have been proposed as being the most relevant for protein-receptor recognition. Our energetic calculations (see materials and methods) confirmed those residues as being the major contributors to the energy of binding of IL-10R1 to IL-10 (S1C Fig).

**Fig 1:**
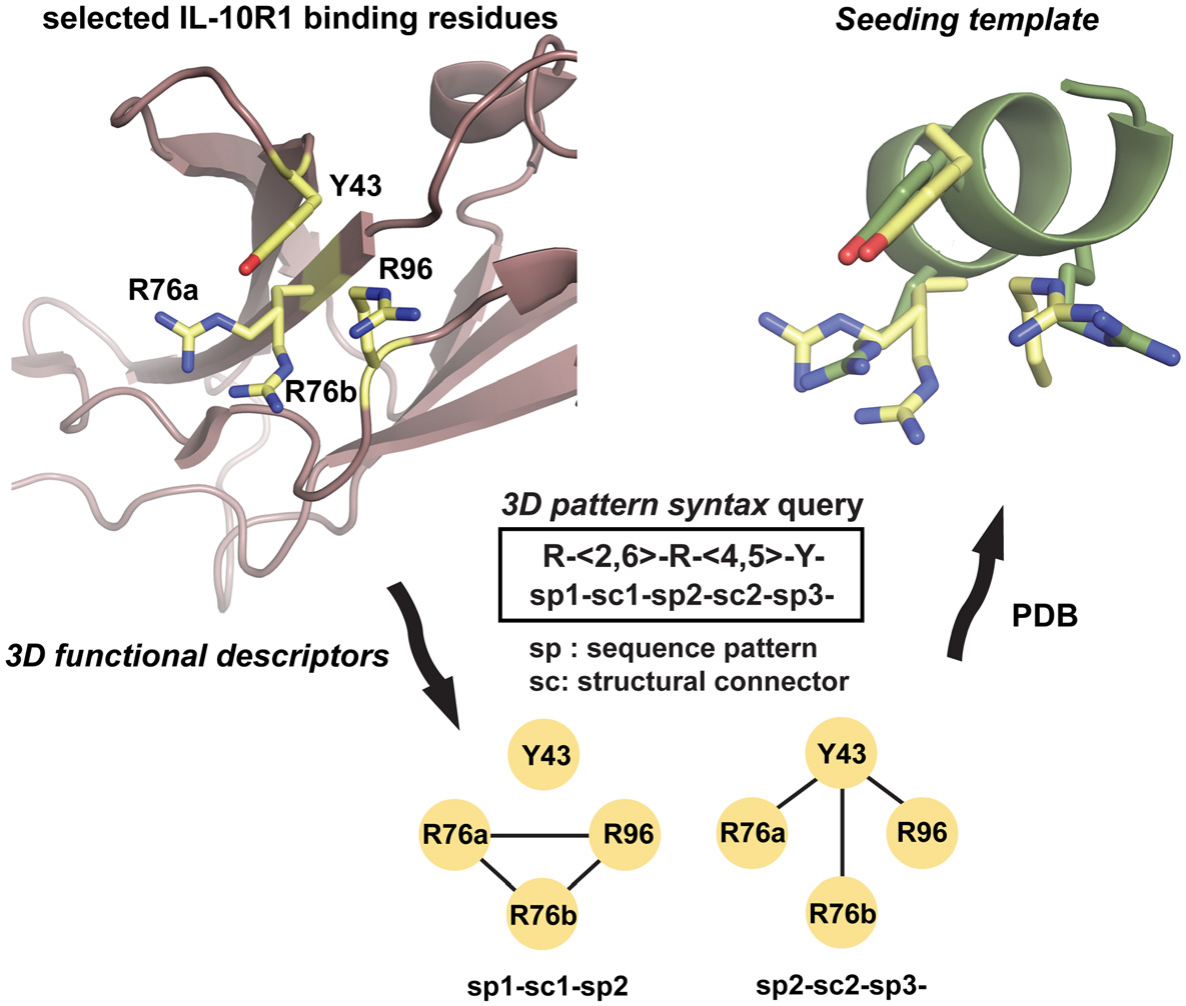
Template-based rescaffolding strategy. *3D functional descriptors* representing the side chain functionalities of selected IL-10R1 binding residues (yellow sticks) are used to define the *3D pattern syntax* query R-<2,6>-R-<4,5>-Y- to search for *seeding templates* in PDB. One of the best hits, used as *seeding template* (2ACA_149-157_, in green), is shown superimposed to the selected IL-10R1 relevant residues for molecular recognition. Molecular images created with PyMOL [29].

In our approach, as strategy to ease the definition of key functionalities in the targeted binding site, we downsized the number of residues to be taken into account by a selection procedure based on: i) calculated residue binding energy contribution, ii) interfacial hydrogen bond network with IL-10, and iii) proximity in space. Based on these criteria, residues IL-10R1_Y43_, IL-10R1_R76_ and IL-10R1_R96_ (S1 Fig), which exhibited top binding energies, an extensive interfacial hydrogen bond network with IL-10 and were disposed in close proximity in space (all within 12 Å), were considered as key functionalities for our *seeding template* search. Though downsizing the large binding epitope to three residues would imply at first none or considerably low binding affinity of any *seeding template* molecule containing them, the geometric and functional constraints demarcated by the side chains of the selected residues would constitute the minimal *3D functional descriptors* to generate regular expressions signifying relevant functionalities of the targeted recognition site to be mimicked (Fig 1).

### 3D Regular expressions describing minimal 3D functional descriptors

The task of searching through repositories of protein structures is considerably more difficult than searching through protein sequence databases. The overall amount of flexibility in protein structures varies considerably, with certain substructures remaining relatively rigid, while others undergo considerable natural variation. Specifying a motif as a specific spatial arrangement of residues has certain weaknesses: firstly, in the computational difficulties of identifying matches, and secondly, in allowing enough flexibility in the pattern so that it can match a broad and meaningful set of structures. The first issue has been addressed in a number of studies such as the template library TESS by using geometric hashing techniques [30] or Jess, which builds on the previous approach by using a back tracking algorithm [31]. Flexibility in the geometric constraints has been previously included as an overall threshold in the accepted RMSD between template and matches structures [32–38]. Our approach to the protein structure search problem has been to develop a procedure that allows us to define structural relationships between flexible sequence patterns. For this, we have taken the commonly used sequence-based PROSITE syntax [22, 23] as bases to formulate a *3D pattern syntax*, which allows a minimalist representation of relationships in 3D space among functionalities without the necessity of introducing any template coordinates as input file (see materials and methods for details).

The above-mentioned *3D functional descriptors* and the geometric relationships among them has been used to generate the 3D syntax query R-<2,6>-R-<4,5>-Y-, which incorporates the selected key functionalities distributed in 3D as desired. Functionalities (side chains) are specified in our search interface as pseudo-points [39]. In the 3D syntax query, R-<2,6>-R describes a range of distances (2 to 6 Å) covering all possible spatial relationships among the side chains of IL-10R1_R96_, IL-10R1_R76a_ and IL-10R1_R76b_ (being R76a and R76b the two conformers found for residue R76 in equal occupancy in the crystal structure). The relation between the functionalities of the side chains of IL-10R1_R96_ and IL-10R1_Y43_, or between one conformer of IL-10R1_R76_ and IL-10R1_Y43_ are described by R-<4,5>-Y. Finally, the hyphen character “-”at the end of the R-<2,6>-R-<4,5>-Y- query specifies a cyclic pattern that reinforces tyrosine functionalities in a way that should be placed in space between the two arginine functionalities already described by the previous syntax elements (Fig 1).

### Search and identification of seeding templates

Our approach uses a 3D pattern query and a search algorithm to scrutinize the PDB. The millions of atomic coordinates collected in the PDB (*ca*. 115.000 experimentally obtained macromolecular structures) and organized in different 3D topologies accommodating endless combinations of functionalities are exploited to identify small regular architectures matching the selected *3D functional descriptors* (see materials and methods for details).

The scrutiny of the PDB by our search engine with the query R-<2,6>-R-<4,5>-Y- threw off a total of 102 matches to our *3D functional descriptors* (S1 Table). Only those hits containing the desired descriptors in a well-defined or regular secondary structure were considered. Furthermore, we selected the shortest architectures (up to 10 residues long) and, by rejecting those that were redundant, we were left with seven candidate structural motifs (Table 1). Interestingly, these contained helical structures, indicating that a helix-like architecture could be considered as a suitable scaffold to achieve an appropriate 3D disposition of the required functionalities. Alpha-helical scaffolds are very attractive from the design point of view as they can be easily stabilized by chemical modifications and, furthermore, they offer many possibilities for substitutions [8, 10, 11]. The selected seven structural motifs were manually superimposed on the X-ray structure of the IL-10/IL-10R1 complex such that a maximal overlap of their functionalities with the selected IL-10R1 binding residues was achieved (Fig 1). Those motifs with best atomic overlapping, 2ARZ_105-109_, 1ZYL_127-131_ and 2ACA_151-155_, were selected. These superposition models were used to analyze which motif would provide the possibility of introducing additional functionalities such that they would engage in the maximum number of complementary interactions with IL-10. In this line, we adopted a straightforward strategy considering the possibility to elongate the alpha helix at the N- and C-termini. 1ZYL_127-131_ and 2ACA_151-155_ were disposed longitudinally with respect to the protein-receptor interface, whereas 2ARZ_105-109_ was transversally oriented (S2 Fig). The longitudinal orientation was able to provide a larger number of interactions with the protein when considering the N- and C- terminal elongation of the helical scaffold. Therefore, the 1ZYL and 2ACA structural motifs were taken into account with two and five additional residues at the N- and C-termini, respectively, which were already in helical conformation in their respective PDB structure. The resulting dodecameric helical scaffold X_1_X_2_R_3_Y_4_X_5_X_6_R_7_X_8_X_9_X_10_X_11_X_12_ (being X any amino acid and underlined positions those representing the 3D functional descriptors) was considered as *seeding template* for further design purposes. Its model in complex with IL-10 was used to investigate in atomic detail those positions that would allow structure-stabilizing chemical modifications excluding positions 3, 4 and 7, which resemble the relevant functionalities of IL-10R1 for IL-10 recognition.

**Table 1:** Residues in PDB matching the *3D pattern syntax* query R-<2,6>-R-<4,5>-Y-. They are underlined in their corresponding PDB structural motifs.

PDB ID (Å)	Residues matching query	Structural motif
2ARZ (2.0)	R_108_/R_105_/Y_109_	_105_-RYYRY-_109_
2G2X (2.3)	R_39_/R_35_/Y_32_	_32_-YQARNFLR-_39_
1UFA (2.2)	R_86_/R_83_/Y_79_	_79_-YAKDRLER-_86_
1XWM (2.5)	R_188_/ R_192_/Y_189_	_188_-RYIER-_192_
1ZYL (2.8)	R_131_/R_127_/Y_128_	_127_-RYLGR-_131_
2ACA (2.2)	R_155_/R_151_/Y_152_	_151_-RYRER-_155_
3KH5 (2.1)	R_76_/R_71_/Y_67_	_67_-YNLIREKHER-_76_

### Rational design strategy and experimental validation

#### Structure-based design of first generation IL-10R1 mimetics

Peptides of less than 15 residues derived from sequences present in protein helical domains usually lack such conformation when they are taken out of the stabilizing environment of the protein. Several approaches have been developed to stabilize short peptides into alpha helical conformations. The formation of covalent linkages between adjacent residues has been one of the preferred methodologies [3–5, 8, 10, 11]. In this regard, lactam bridges between the side chains of lysine and aspartic acid in positions *i*,*i* + 4 have been shown to stabilize most efficiently short synthetic peptides into an alpha-helical structure [40]. We therefore adopted a lactam bridge design strategy. Our 12-mer *seeding template* X_1_X_2_R_3_Y_4_X_5_X_6_R_7_X_8_X_9_X_10_X_11_X_12_ (*vide supra*) offers the possibility to accommodate two *i*,*i* + 4 lactam bridges, which, in order to fix the desired conformation of positions 3, 4 and 7 with respect to IL-10 (*i*.*e*. in two consecutive helical turns), could be introduced separately or consecutively: [K_1_X_2_R_3_Y_4_D_5_]X_6_R_7_[K_8_X_9_X_10_X_11_D_12_] and [K_1_X_2_R_3_Y_4_D_5_][K_6_R_7_X_8_X_9_D_10_]X_11_X_12_, respectively (square brackets represent lactam bridges between lysine (K) and aspartic acid (D) side chains) (S3A and S3B Fig). In the first scenario, the twelve residues would be constrained in helical conformation. In the second, only residues 1 to 10 would be constrained. Nevertheless, the helical conformation could be extended beyond these constraints at least by one or two more residues [41]. Based on our 3D molecular models, the first option would offer only position 11 for introducing additional H-bond interactions with IL-10, whereas in the second option positions 8 and 11 would be available. In addition, our models suggested that the region of IL-10 interacting with the residue in position 11 (loop AB) would require certain flexibility in its counterpart. Therefore, two consecutive *i*,*i + 4* lactam bridges were introduced in the scaffold resulting in a bicyclic molecule, which was then subjected to a per residue structure-based mutagenesis process in order to achieve best binding complementarity to IL-10. Here, substitutions at positions 3, 4, 7, 8 and 11 pointed towards IL-10, whereas residues at positions 9 and 12 did not establish contacts with the protein, and they were therefore maintained unaltered as in the corresponding PDB template structure (2ACA_149-160_). Position 12 could be used to enhance helicity in the C-term. Following this rationale, a lysine residue was introduced at position 8 to favor interactions with IL-10_D44_, and position 11 was used to introduce an arginine side chain for interactions with IL-10_D44_ and IL-10_Q42_ (S3C Fig). In order to analyze the possibility that the four positive charges so-far introduced in our bicyclic template could engage in non-specific electrostatic interactions at other regions of IL-10, we performed energy interaction calculations with a sp^2^ amine NH_2_ cation and a sp^3^ amine NH_3_ cation chemical probes by using GRID [42]. The most favorable energy was obtained at the predicted binding region for our template (S4A Fig), suggesting that the selected topology of charged residues would confer sufficient binding specificity to the designed molecule.

**Fig 2:**
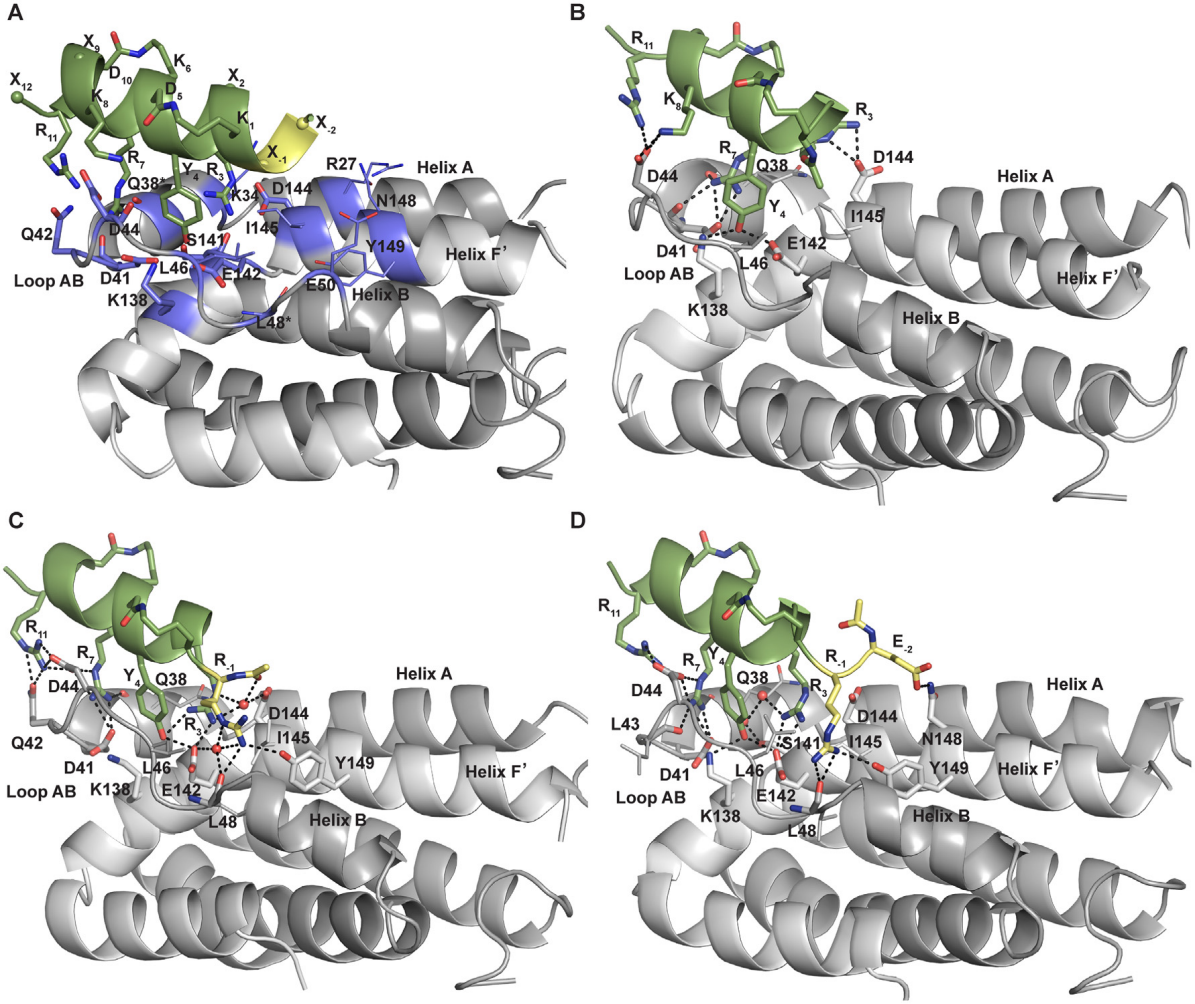
Structure-based design rationale. (A) Atomic representation of the model of the complex between IL-10 (gray, PDB ID 1J7V) and the helical scaffold used in the rationale for the design of IL-10R1 mimetics (green, *N*-terminal functionalization in yellow). For clarity, no side chains shown for IL-10_Q38_ and IL-10_L48_. (B) Snapshot of complex IL-10/M1 (at 2.9 ns from 10 ns MD simulation). (C) Snapshot of complex IL-10/M4 (at 10 ns). (D) Snapshot of complex IL-10/M6 (at 3.6 ns). Residues involved in recognition are shown in sticks and colored by atom type (IL-10 highlighted in violet in A). Red spheres represent interfacial structural waters observed during MD simulations. Intermolecular H-bonds are depicted by black dashed lines. Figure generated in PyMOL [29].

At this first stage, our rational design strategy also included additional strategic residue modifications directed towards the experimental evaluation of ligand binding at the protein targeted site by means of fluorescence spectroscopy (*vide infra)*. A tryptophan or its 5-hydroxy derivative were introduced at position 2 of our ligands as labels to enable monitoring of protein complex formation. Furthermore, in order to enhance the sensitivity of tryptophan fluorescence measurements upon ligand binding at the targeted site, our rationale also made use of a mutation of cysteine to tyrosine in the protein binding region (*i*.*e*. at position 149, see below and Fig 2 for details). Thus, we could anticipate that, in case of ligand binding to the protein in the targeted site, the tryptophan emission produced would be enhanced by an energy transfer from the neighbor protein residue Y149. Finally, our template was acetylated and amidated in its N- and C-termini, respectively. The resulting IL-10R1 mimetic molecule Ac-[KW_2_R_3_Y_4_D][KR_7_K_8_VD]R_11_A-NH_2_ (**M1**, Table 2, Fig 2) was refined in complex with IL-10 using MD simulations (see materials and methods for details). The mimetic-protein binding free energy (ΔG_M-P_) was estimated with the MM-PBSA method and indicated favorable electrostatic and van der Waals contributions to the binding [43, 44] (Table 2). Hydrogen bonding between both molecules was evaluated (S2 Table). In the refined model, R_3_ participated in a hydrogen bond with IL-10_D144_, Y_4_ with IL-10_K138_ and IL-10_E142_, R_7_ with IL-10_Q38_ and IL-10_D41_. K_8_ and R_11_ formed hydrogen bonds with IL-10_D44_. Furthermore, the aliphatic moieties of the R_3_, Y_4_ and R_7_ side chains of the mimetic showed important van der Waals contributions with the side chains of IL-10_I145_ and IL-10_Q38_. The relevance of mimetic residues Y_4_, R_5_ and R_7_ in binding to IL-10 was further investigated by MM-PBSA alanine mutagenesis [43–45]. The substitution of these residues by alanine revealed a considerable unfavorable effect in binding for all mutants, showing the most dramatic effect for alanine mutation at position 7 (R7A, S3 Table).

**Table 2:** Mimetic-protein binding free energy obtained by MM-PBSA [43, 44] and experimental dissociation constants (*K*_d_).^[a]^^,^^[b]^

IL-10R1 Mimetic	Sequence	ΔG_M-P_ (kcal/mol)^[c]^	*K*_d_ (μM)
**M1**	Ac-[KWRYD][KRKVD]RA-NH_2_	-27.1 ± 6.6	> 30^[a]^
**M2**^[d]^	X-[KWRYD][KRKVD]RA-NH_2_	-53.3 ± 9.9	5.0^[a]^
**M3**^[d]^^[e]^	X-[KZRYD][KRKVD]RA-NH_2_	-59.9 ± 9.2	6.7^[a]^
**M4**	Ac-R[KWRYD][KRKVD]RA-NH_2_	-59.2 ± 7.0	8.4^[a]^
**M5**	Ac-EK[KWRYD][KRKVD]RA-NH_2_	-77.9 ± 9.7	0.07 ± 0.02^[b]^
**M6**	Ac-ER[KWRYD][KRKVD]RA-NH_2_	-71.8 ± 8.0	0.04 ± 0.02^[b]^

^[a]^ *K*_d_ values calculated from thermal denaturation data.

^[b]^ *K*_d_ values inferred from ITC thermodynamic data.

^[c]^ ΔG_M-P_ = Mimetic-Protein binding free energy. Entropy contribution to binding was not estimated here.

^[d]^ X = 4-guanidinobutanoyl.

^[e]^ Z = 5-Hydroxy-L-tryptophan.

#### Experimental validation of first generation IL-10R1 mimetics

**M1** was chemically synthesized, and its experimental binding affinity towards IL-10 was assessed by thermal stability measurements using circular dichroism (CD) [46] and fluorescence [47] (see materials and methods). For these studies, we used the readily available murine IL-10 mutant mIL-10_C149Y_ in which Cys149 was replaced by the corresponding Tyr residue of the human protein, as it was previously shown to enhance protein refolding yield [48, 49]. Furthermore, this tyrosine would enhance the sensitivity of tryptophan fluorescence measurements *(i*.*e*. as stated above, it can act as energy donor for the tryptophan residue introduced as label in the mimetics).

Human and murine IL-10 share high sequence similarity (88%), and the residues forming the mimetic binding site are conserved in both. Furthermore, the selected IL-10R1_Y43_, IL-10R1_R76_ and IL-10R1_R96_ binding residues used for our design are fully conserved in both human and murine receptors. The 3D structure of mouse IL-10 and its receptor are not available. Therefore, in order to investigate the suitability of the use of mIL-10_C149Y_ for our studies, and for comparison purposes between the murine and human proteins, atomic models of mIL-10_C149Y_ and mIL-10R1 were built by applying homology modeling techniques (see materials and methods for details and S5 Fig). The obtained 3D mouse models were analyzed in detail in context of the crystal structures of human IL-10 and its complex structure with IL-10R1 to investigate any structural differences, in particular at the recognition site (S5 Fig). In mIL-10_C149Y_, most residues involved in the mimetic binding interface are conserved in sequence and 3D space disposition. Residues mIL-10_Q32_ (R in human, helix A), mIL-10_T39_ (M in human, helix A) and mIL-10_T49_ (K in human, loop AB), although not conserved, were not exposed to the mimetic and, therefore, were not expected to interfere with its binding. Residue mIL-10_N141_ (S in human IL-10, helix F’) is located at the recognition site of the conserved receptor residues mIL-10R1_R76_ and mIL-10R1_R96_, and it could therefore establish similar interactions as in human IL-10. mIL-10_I46_ (L in human IL-10, loop AB) is semi-conserved and would participate in van der Waals interactions with mIL-10R1_Y43_ as in the human protein-receptor complex. mIL-10_D50_ (E in human, helix B) is also semi-conserved and could participate in additional interactions with the N-terminally elongated IL-10R1 mimetics. Based on the sequence and structural similarity of the mimetic recognition site in the human and mouse proteins and the receptors, we concluded that mIL-10_C149Y_ could appropriately serve our experimental needs, and that the designed IL-10R1 mimetics should also bind to it as predicted for the human protein.

**Fig 3:**
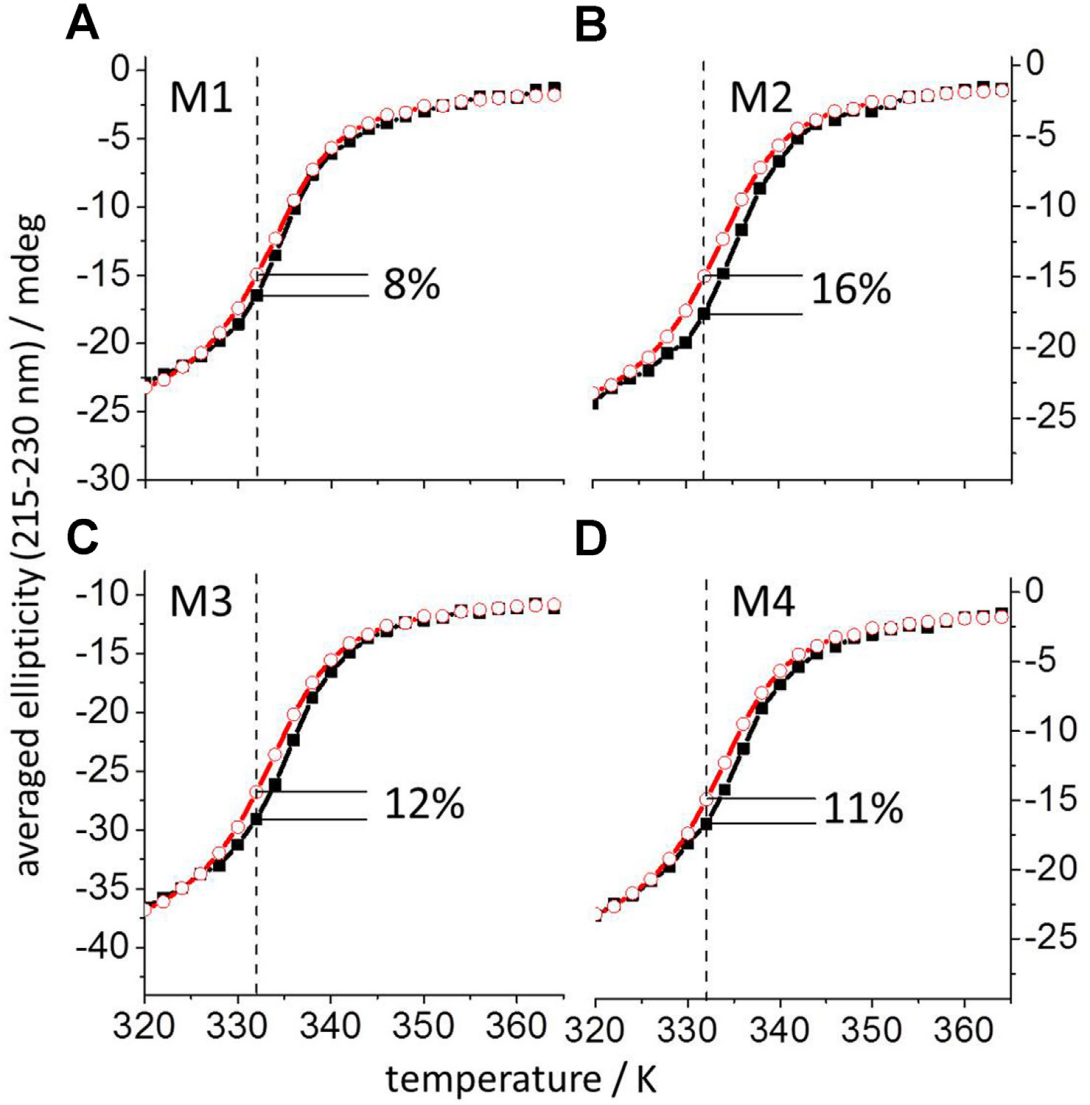
Temperature denaturation curves obtained by CD for mIL-10_C149Y_ in complex with IL-10R1 mimetics M1-M4. The average thermal denaturation CD signal in the 215 to 230 nm range is plotted as a function of temperature for mIL-10_C149Y_ alone (2 μM, red open circles) and for mIL-10_C149Y_ in the presence of each mimetic M1-M4 (4.5 μM, black filled squares) in panels A-D, respectively. The obtained mIL-10_C149Y_ CD data agree with those published earlier for human and murine IL-10 [50]. The highest amount of native protein structure binding to each of the mimetics M1-M4 was observed at 332 K (dashed lines) and is given in each corresponding panel as percentage of the total change in the CD-signal.

**Fig 4:**
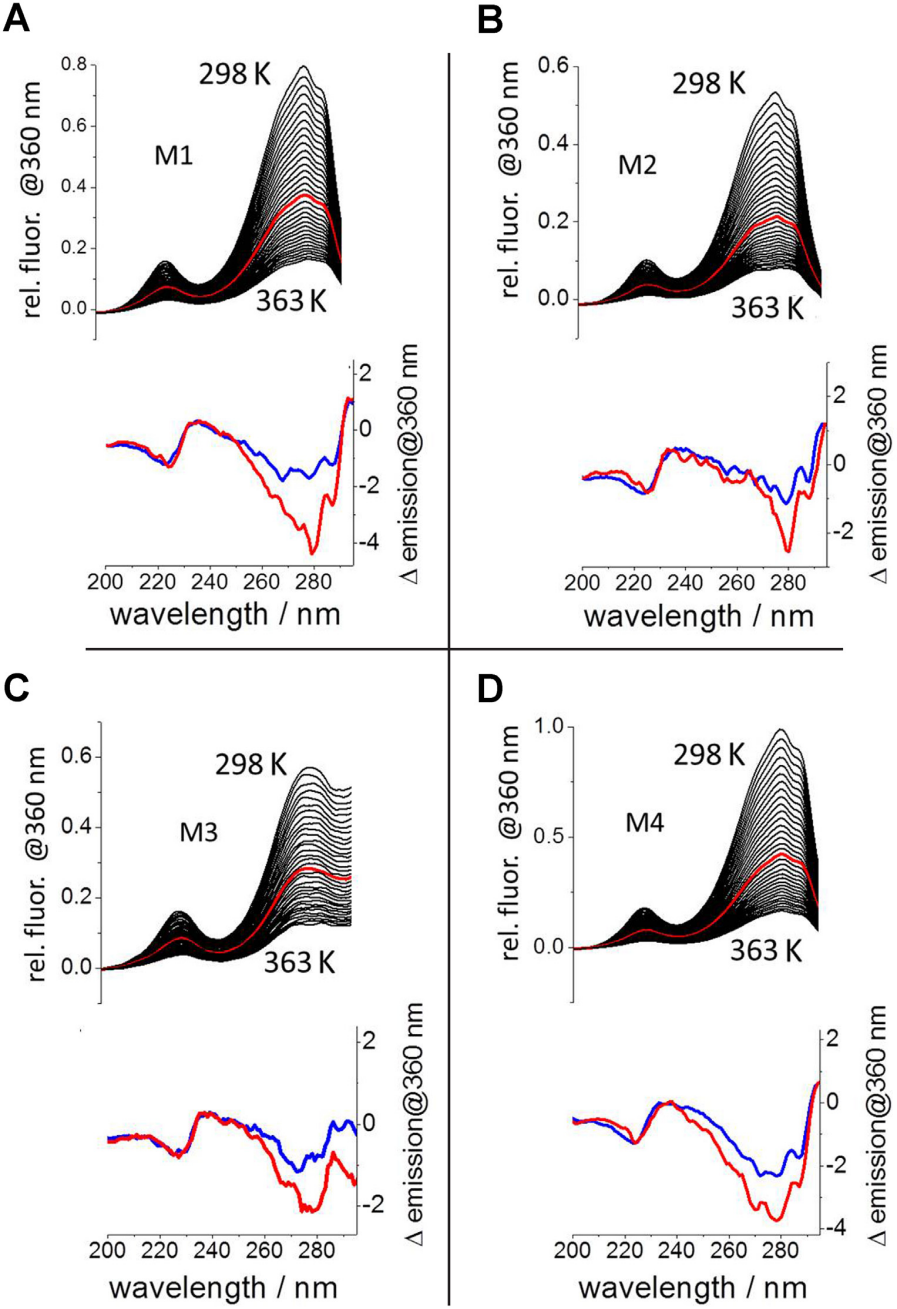
Temperature-dependent excitation spectra of the tryptophan in mimetics M1-M4. Upper plots A-D: Mimetic tryptophan emission (excited between 200 and 290 nm) measured at 360 nm for the temperature range between 298 K and 363 K for each mimetic M1-M4 in the presence of mIL-10_C149Y_. The excitation spectra at 332 K are highlighted in red. Lower plots A-D: Subtraction of the excitation spectra at 298 K from those at 332 K (red) in comparison with the spectra obtained in absence of mIL-10_C149Y_ (blue).

The experimentally estimated binding affinity shows that **M1** binds to the protein (Table 2, Figs 3 and 4). Complex formation between **M1** and mIL-10_C149Y_ was determined by thermal denaturation using CD and the well-known negative ellipticity of IL-10 between 200 to 300 nm [40]. The average CD signal in this range of wavelengths was measured as a function of temperature between 298 and 363 K. Fig 3A shows a decrease of negative ellipticity, which exhibits a sigmoidal shape typical of thermally induced protein unfolding. The obtained curves shift to higher temperatures in the presence of **M1**. The shift is due to a lower degree of unfolded protein at any temperature in the presence of mimetic **M1** as compared to the denaturation of mIL-10_C149Y_ alone. The largest difference was observed at 332 K and is expressed in percentage of the total unfolding signal (obtained after subtraction of the initial and final linear segments of the curves). At 332 K, the presence of **M1** increased the native protein structure by 8%.

Simultaneously with the thermal stability CD-signals, the excitation spectrum of the tryptophan residue strategically introduced at position 2 in **M1** was measured. The results obtained provided an additional assessment of complex formation between **M1** and mIL-10_C149Y_ (Fig 4A). We observed that the emission at 360 nm decreased with increasing temperatures, leading to the decline of the excitation spectra between 200 and 290 nm (Fig 4A upper plot, excitation spectra at 332 K highlighted in red). The underlying spectral differences are clearly appreciated in the representation of the subtraction of the excitation spectrum at 298 K from that at 332 K in the presence and absence of mIL-10_C149Y_ (Fig 4A lower plot, red and blue line, respectively). The enhanced temperature sensitivity of tryptophan fluorescence obtained in the presence of mIL-10_C149Y_ supports a binding interaction. The increased fluorescence emission is explained by a restriction in movement of the indole ring at the binding interface and the energy transfer from Y_149_ in mIL-10_C149Y_. Both factors would vanish upon thermal denaturation, leading to the observed larger drop in fluorecence excitation in the presence of mIL-10_C149Y_. These data therefore support the interaction of **M1** with mIL-10_C149Y_ as derived from the CD-denaturation curves.

#### Rational optimization of first generation IL-10R1 mimetics and experimental validation

Two iterative cycles of *in silico* optimization and experimental validation were carried out with the purpose of increasing the interactions between **M1** and IL-10 and, therefore, obtain potent IL-10R1 mimetics.

By comparing the structures of the refined model complex IL-10/**M1** and the X-ray structure of IL-10/IL-10R1, it was envisaged that N-terminal functionalization of **M1** could potentially contribute with additional complementary interactions to the protein. Indeed, in the MD simulation of the IL-10/IL-10R1 complex the following interactions were observed: IL-10R1_N73_/IL-10_E50_, IL-10R1_N73_/IL-10_L48_, IL-10R1_D100_/IL-10_R27_ and IL-10R1_E101_/IL-10_K34_. Thus, based on the structure of the refined model of the **M1**/IL-10 complex (Fig 2A and 2B) and taking into account the interaction energies obtained with the sp^3^ and sp^2^ amine cation chemical probes (*vide supra*), two new mimetics (**M2**, **M3**; differing in tryptophan *vs*. its 5-hydroxy derivative in position 2) were designed by N-terminal functionalization with 4-guanidinobutanoyl, providing a positively charged H-bond donor for potential interaction with the negatively charged side chain of IL-10_E50_ (helix B). The same holds true for the extension of the N-terminus of **M1** by an arginine (**M4**) or lysine, which could offer stabilizing interactions with the backbone of IL10_L48_ (loop AB) and/or the side chain of IL-10_Y149_ (helix F’). The binding affinities of IL-10R1 mimetics **M2**, **M3** and **M4**, determined as described for **M1** (*vide supra*), showed a considerable improvement (Table 2, Fig 3).

As for **M1**, the obtained fluorescence data for mimetics **M2**-**M4** clearly revealed enhancement of tryptophan emission when the mimetics were bound to the protein (Fig 4B–4D). As described above, this is explained by the immobilization of the tryptophan of the mimetics and the energy transfer from the protein tyrosine in position 149 upon complex formation, which support the protein-mimetic interaction at the expected binding site. This piece of evidence supported our design rationale.

**Fig 5:**
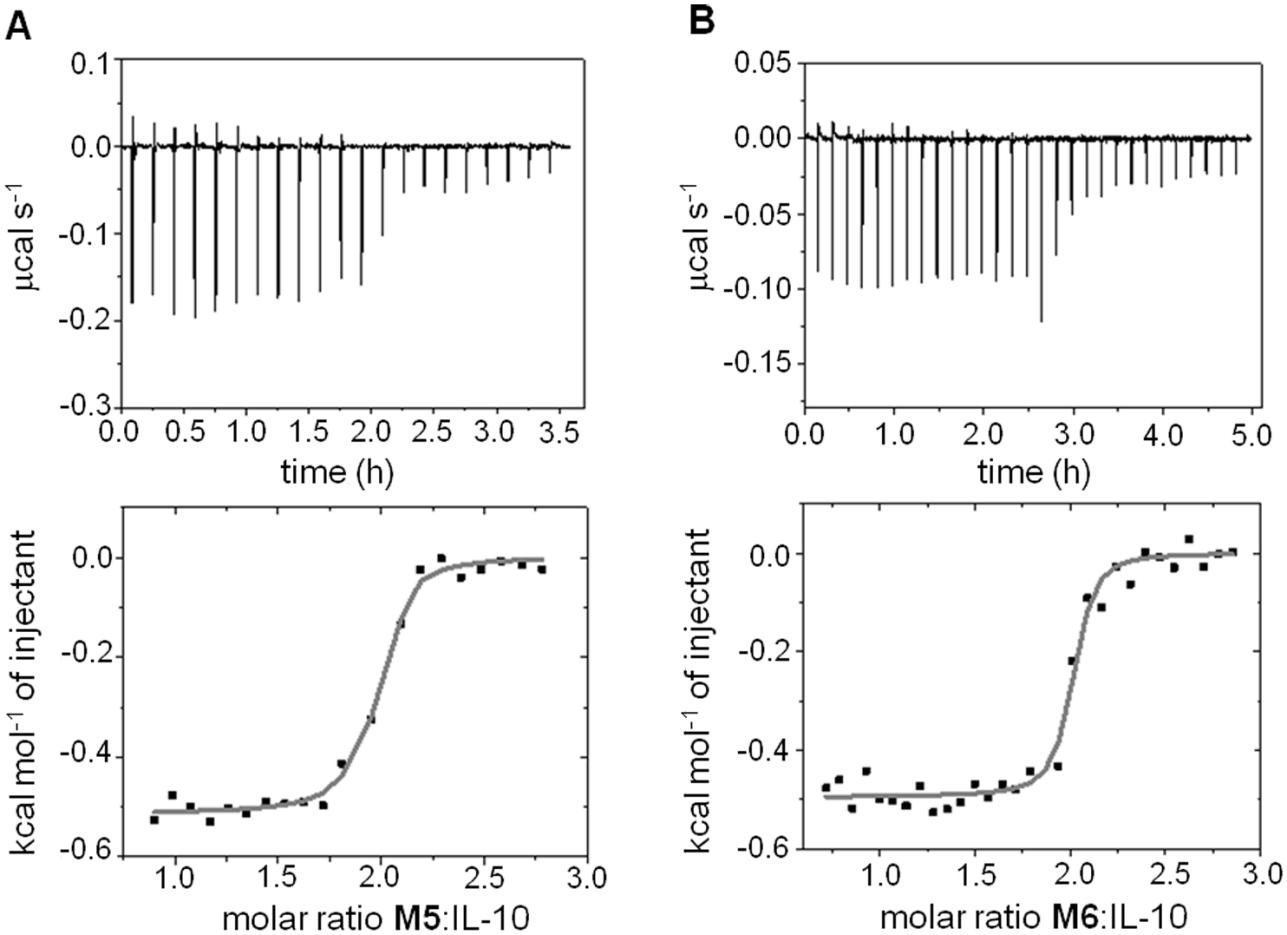
Isothermal titration calorimetry. (A) mIL-10_C149Y_ (43 μM) with M5. (B) mIL-10_C149Y_ (25 μM) with M6. Upper and lower panels show the raw data and integrated heat changes with the corresponding fitted binding curves based on a single site model, respectively.

Further ligand optimization was accomplished by the introduction of an additional N-terminal H-bond acceptor such as Gln or Glu (**M5**, **M6**), which would allow interactions with the cationic side chains of IL-10_R27_ and IL-10_K34_ (helix A) or with IL-10_N148_ (helix F’) (Fig 2A). Favourable energies obtained for the interaction of IL-10 with an aliphatic carboxylate chemical probe [42, 51] supported this rationale. Furthermore, residues IL-10_L46_ (loop AB) and IL-10_I145_ (helix F’) would additionally contribute with van der Waals interactions with the aliphatic moieties of the side chains of the residues introduced at the N-teminus. The computationally obtained binding free energies and the experimental *K*_d_ estimates (Table 2) showed correlation and a clear improvement in binding due to N-terminal functionalization, reaching nanomolar affinities for **M5** and **M6** as determined in more detail by isothermal titration calorimetry (ITC, see materials and methods) (Fig 5). Furthermore, these measurements supported the expected stoichiometry 2:1 mimetic:dimer-protein binding ratio as previously observed for the IL-10R1/IL-10 complex [28].

All in all, the step-wise increase in binding obtained computationally and experimentally for the designed molecules along our iterative rational strategy support the design rationale.

The analysis of the interfacial H-bond formation observed along the corresponding MD trajectories of the protein-mimetic complex series (see materials and methods) corroborated the envisaged interaction patterns. First, the introduction of new H-bond donors at the N-terminus of **M1** led to complementary interactions with acceptor groups of protein residues L48, E50, E142 and Y149 (**M2**-**M4**, S2 Table, Fig 2C). Second, the N-terminal extension by an additional residue acting as an H-bond acceptor (**M5** and **M6**) allowed interactions with the donor groups of protein residues K34, Q38 and N148 (S2 Table, Fig 2D). The data obtained from the MD trajectories were also used to analyze the dynamic behaviour of solvent, in particular those water molecules that were participating in the binding of the mimetics to the protein. In the case of mimetics **M3** and **M4**, a well-defined water molecule was observed bridging by H-bond their guanidinium groups in X and R_-1_, respectively, with the protein residues L48 and E142 (S2 Table). In **M5**, a water molecule located in the mimetic-protein interfacial core was observed to bridge residue Y_4_ and protein residues D41 and S141. Bridging water molecules were also involved in interactions between residue R_3_ in **M6** and protein residue S141 (S2 Table, Fig 2D). These observations highlight the relevance of interfacial solvent for the protein-mimetic recognition, as demonstrated for other PPIs [52–54], and the importance of the solvation effect for ligand recognition [55]. Furthermore, per residue energetic contributions obtained from MM-PBSA indicated favourable hydrophobic contributions. The most relevant van der Waals interactions were observed between the aliphatic moieties of K_-1_, R_-1_, X, R_3_, Y_4_, K_8_ of **M2**-**M6** and the protein residues D44, L46, D144 and I145 (S6 Fig). The aliphatic moiety of residue R_7_ in the mimetics also contributed to hydrophobic interactions with the protein residues Q38 and Q42. The results obtained from the MM-PBSA alanine scanning carried out on the residues R_3_, Y_4_ and R_7_ in mimetics **M4** and **M6** were consistent with the decrease in protein binding observed for these mutations in **M1** (S3 Table). Mimetics **M2**, **M3** and **M4** incorporating one H-bond donor at the N-terminus of **M1** showed *K*_d_ values below 10 μM, reflecting an enhancement of binding higher than 6-fold in the best case with respect to the lead **M1**. The maximal binding enhancement was obtained for **M5** and **M6**, which showed binding affinities values in the nanomolar range, corresponding to a more than 70-fold increase with respect to **M2**-**M4**, and at least more than 400-fold with respect to **M1**. Thermodynamic parameters determined from ITC measurements for the most potent designed IL-10 ligands (**M5** and **M6**) indicated that their free energies of binding were mostly driven by a favourable entropic component (TΔS 9.5 kcal/K·mol) with a weak favourable enthalpic term (ΔH -0.5 kcal/mol). The observed favourable entropic contribution was attributed to the intrinsic rigid structural nature of the protein, the pre-organized helical nature of the mimetic, as described for other structurally constrained systems [56, 57], the van der Waals interactions established between several protein and mimetic residues, and the presence of structural bridging waters involved in protein-mimetic binding. The latter was further investigated by analyzing the overlap of the interaction energy maps obtained for the unbound IL-10 (PDB ID 2ILK (1.6 Å) [27]) with a GRID “solvent” water and a “solvent exclusion” sp^3^ carbon chemical probes [42, 54]. A favourable energy contribution for solvent was indicative of the presence of three water molecules at the mimetic binding site, which, interestingly, overlapped with three structural waters present in the crystal structure of the free IL-10. Furthermore, the “solvent exclusion” probe provided one of the largest energetic contributions in the same region where these waters are located (S4B Fig). This analysis further indicates an entropic contribution resulting from the release of structurally bound water molecules upon mimetic binding.

### Conclusions

The plethora of information currently contained in the three-dimensional protein chemical space available in the PDB offers a great ground for identifying defined topologies containing endless combinations of functionalities in 3D space. This pool of millions of atomic coordinates topologically arranged in 3D space can be exploited to facilitate rescaffolding strategies for the design of natural-like chemical architectures to tackle challenging protein-protein interactions. A customized regular expression syntax describing particular 3D arrangements of functionalities relevant for recognition within discontinuous unstructured protein regions has been developed in order to scrutinize such available structural information of the PDB for matches to regular structural frameworks already present in nature. The straightforward identification of adequate *seeding* structural templates with the desired 3D arrangements of functionalities using this minimalistic approach has been key for a plainspoken and successful *de novo* design of a new class of potent molecules that mimic unstructured and discontinuous regions of receptor IL-10R1 responsible of its high affinity for IL-10. The molecular rational design approach described here constitutes a step forward towards tackling challenging protein-protein interfaces because it smoothes existing limitations for mimicry of interactions spanning large and poorly structured binding epitopes in a sequence-connectivity independent manner.

## Materials and Methods

### 3D Pattern regular expression methodology

Our methodology is inspired on the well-established PROSITE syntax commonly used in protein sequence analysis [22, 23], which consists of a collection of pre-defined sequence profiles (defining protein families or functional motifs) that is used to find matches in protein sequence databases. In particular, our methodology involves two technical contributions: a 3D pattern syntax and a structural pattern *search algorithm*.

**Fig 6:**
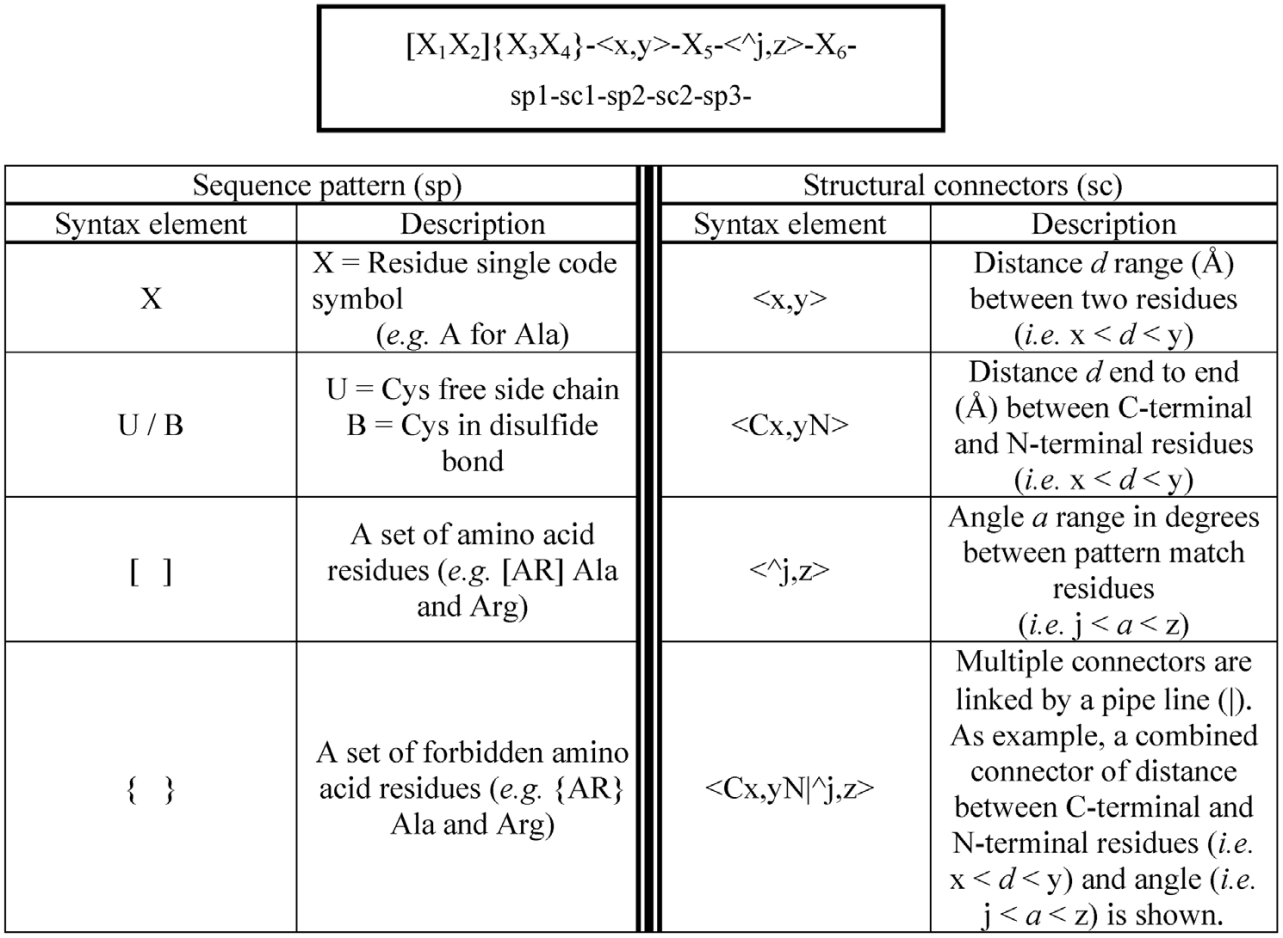
*3D pattern syntax*: Sequence patterns and structural connectors.

- The ***3D pattern syntax*** developed here uses the PROSITE pattern syntax as starting point. It contains *sequence patterns* defining functionalities at residue level, and a variety of *structural connectors* defining distance and angle ranges and their inter-relationships (Fig 6). This syntax allows multiple *sequence patterns* to be joined using flexible *structural connectors*. The combination of all these syntax elements confers flexibility in the definition of sequence and structural constraints representing desired functionalities in each individual syntax query.

A *sequence pattern* consists of a definition of one or a combination of amino acids. The later is represented with square brackets “[]” for allowed residues and curly brackets “{}” for forbidden residues. The geometric relationship between *sequence patterns* in three-dimensional space is established by using geometrical descriptors, which we call *structural connectors*. A *structural connector* is defined in terms of a range of values: i) distances (in Angstroms) between the alpha carbons or a functional center of the side chain of amino acids, the latest denoted as pseudo atoms roughly equivalent to the center of mass of the side chain and inspired in previous work of Bahar *et al* [39]; ii) angles (in degrees) formed by the corresponding peptide sequence backbones. The use of pseudo-points represents an advantage over calculating all pairwise distances, which would considerably increase the calculation time (about 16 times). Distances between alpha carbons or pseudo-points and angles between alpha carbons are specified in the search interface by selecting either alpha-carbons or pseudo-points for each search. A *structural connector* is denoted by a pair of angle brackets “< >”. Structural constraints can be combined by introducing a pipe-line “|” between connectors. The connection between two *sequence patterns* and a *structural connector* is defined by a hyphen character (*i*.*e*. “-“). A cyclic pattern is built by adding this hyphen at the end of the last sequence pattern or structural connector.

A particular *3D pattern syntax* query defining geometric relationships of desired functionalities is used as input by our structural pattern search algorithm.

- The *structural pattern* ***search algorithm*** operates across multiple stages designed to minimize the computational overhead of querying large protein structure datasets (*i*.*e*. the Protein Data Bank PDB) directly.

1. First, and as a pre-filtering step, the search engine utilizes a dataset of sequences (extracted from the 3D structures to be analyzed; *i*.*e*. PDB) for candidate hits matching. A candidate hit is any protein sequence that has each of the specified sequence elements or sub-sequences in the pattern. Sub-sequences should not overlap.
2. A data-structure of all available sub-sequences in a given protein (often there are multiple matches for each sub-sequence) is built by considering all hits obtained in the previous step. This data-structure is then used in the next stage.
3. The engine verifies that there is at least one set of sub-sequences that matches the required structural constraints specified in the *3D pattern syntax* query. As the combinatorial space can be extremely large, the engine makes use of a recursive backtracking algorithm to minimize redundant computation and permit early termination when one of the required sub-sequences has been found to fail all structural constraints.

As multiples matches could be found for the same structure, and in order to improve computational efficiency and to avoid large numbers of very minor variations, we add the additional constraint that multiple matches in the same structure cannot overlap any components. In addition, we run the structural pattern search algorithm over a redundancy reduced data set. When matches are found, we are able to manually explore similar structures for equivalent matches.

All algorithms developed to interrogate the protein structure space in a systematic manner searching for patterns in databases have to make a trade-off between three key areas: 1) The pattern expressivity (*i*.*e*. range of functional motifs that can be captured), 2) The ease of pattern definition, 3) The computational efficiency. In our approach, we scarify some of the first feature in order to achieve better performance on the latter two. Our methodology presents a number of key differences with other comparable computational techniques such as RAMOT-3D [36], ASSAM [37] and IMAAAGINE [38] and overcomes some of their limitations. Typically, in these tools the search syntax does not allow for variations in the identities of residues in particular positions as our does. In our case, the patterns are inspired on the PROSITE syntax and therefore defined using amino acid sequence elements. As result easy and flexible patterns can be designed instead of using graphs. Furthermore, our patterns can have arbitrary numbers of amino acids containing distance and angle tolerances described for each connection instead of being globally defined. Besides, the chemical properties in our patterns are defined in our case only through sets of amino acids and not via many pre-defined sets.

### Computational structure-based design and simulation

We used the 3D crystal structure of human IL-10 in complex with IL-10R1 for our calculations (PDB ID 1J7V, 2.9 Å [28]). This structure contains a protein dimer and two receptor molecules. Due to the two-fold axis symmetry in this complex, only one of the two IL-10 domains and a receptor molecule were considered for our studies (S1 Fig).

#### Analysis of relevant binding residues in the IL-10/IL-10R1 interface

To determine the receptor residues being the major contributors to the protein binding energy, molecular dynamics calculations were carried out with the IL-10/IL-10R1 complex. AMBER11 and AMBER12 packages [58] were used for the simulations, and the MM-PBSA method [43, 44] as implemented in AMBER11 was used to obtain per residue energy contributions (S1C Fig). The ff99SB force field [59] and standard protocols as implemented in the AMBER11 and AMBER12 packages were used (see below) [58]. The IL-10/IL-10R1 complex was solvated in a truncated octahedral box of TIP3P water molecules and neutralized with Cl^-^ counterions. Molecular dynamics simulations were preceded by two energy-minimization steps: i) only the solvent and ions were relaxed; ii) the entire system was minimized. The system was then heated up from 0 K to 300 K in 50 ps. Weak position restraints (10 kcal/mol·Å^2^) were used for the whole system in the canonical ensemble (NVT). Langevin temperature coupling with a collision frequency γ = 1 ps^−1^ was used at this step. The system was equilibrated without restraints at 300 K during 100 ps in the isothermal-isobaric ensemble (NPT) followed of other 100 ps in the canonical ensemble (NVT). The Langevin thermostate and Berendsen barostat under periodic boundary conditions were used during the equilibration steps. After this, a total of 20 ns MD production without restraints was carried out at 300 K in the canonical ensemble (NVT) using the Langevin thermostat. The SHAKE algorithm was used to constrain all bonds involving hydrogen atoms. A time step of 2 fs was used. A cutoff of 8 Å was applied to treat the non-bonded interactions, and the Particle Mesh Ewald (PME) method was used to treat long-range electrostatic interactions. MD trajectories were recorded every 2 ps. Trajectories were visualized with VMD [60] and evaluated in terms of intermolecular H-bonds using the PTRAJ and CPPTRAJ modules implemented in AMBER. The criterion used to consider a dynamic hydrogen bond formation was to be found at least 10% of the simulation time using a distance acceptor-donor cutoff of 3.5 Å and a 120° angle cutoff.

#### Mimetic refinement and analysis

In each step of the lactam bridge design strategy (S3 Fig), a brief minimization was performed with MOE (Amber12:EHT force field and default parameters were used) [61]. Molecular dynamics simulations of the complex of IL-10 with each of the designed IL-10R1 mimetics were performed using the ff99SB force field [59] and standard protocols as implemented in the AMBER11 and AMBER12 packages (see below) [58]. The mimetic residues Lys and Asp involved in lactam bridges were parametrized according to the ff99SB force field, and RESP charges were derived at the HF/6-31G* calculation level using Gausian09 [62–64]. Same strategy was followed for parameters of the chemical groups introduced in the optimization process that were not included in the ff99SB force field. Each protein-mimetic complex was solvated in a truncated octahedral box of TIP3P water molecules and neutralized with Cl^-^ counterions. Molecular dynamics simulations were preceded by two energy-minimization steps: i) only the solvent and ions were relaxed; ii) the entire system was minimized with low position restraints (10 kcal/mol·Å^2^) for the helical backbone section of the mimetic. The system was then heated up from 0 K to 300 K in 20 ps. Weak position restraints (10 kcal/mol·Å^2^) were used for the protein and the mimetic in the canonical ensemble (NVT). Langevin temperature coupling with a collision frequency γ = 1 ps^−1^ was used at this step. Along three equilibration steps of 500 ps each, the helical backbone position restraints and also two distance restraints between protein and mimetic (IL-10_Q38_/**M**_R7_ and IL-10_K138_/**M**_Y4_) were consecutively decreased (10, 5, and 2 kcal/mol·Å^2^, respectively). This was carried out in the isothermal-isobaric ensemble (NPT) using the Langevin thermostate and Berendsen barostat under periodic boundary conditions. The system was further equilibrated during 1 ns without restraints at 300 K under same conditions. After this, a 10 ns MD production was carried out at 300 K in the canonical ensemble (NVT) using the Langevin thermostat. The SHAKE algorithm was used to constrain all bonds involving hydrogen atoms. A time step of 2 fs was used. A cutoff of 10 Å was applied to treat the nonbonded interactions, and the Particle Mesh Ewald (PME) method was used to treat long-range electrostatic interactions. MD trajectories were recorded every 2 ps. Trajectories were visualized with VMD [60] and evaluated in terms of intermolecular H-bonds (including bridging water molecules at the interface between the designed mimetics and IL-10) and RMSD using PTRAJ and CPPTRAJ modules implemented in AMBER. The criterion used to consider a dynamic hydrogen bond formation was to be found at least 10% of the total simulation time (S2 Table). Water molecules connecting functionalities (bridging waters) were also considered for hydrogen bond analysis using a distance acceptor-donor cutoff of 3.5 Å and angle cutoff of 120°. A structural bridging water was considered to be participating in H-bond in protein-mimetic complex when was found at least 20% of the total simulation time (S2 Table). Energy decomposition per residue and binding free energy post-processing analysis of the trajectories (Table 2) were performed in implicit solvent using the MM-PBSA method [43, 44] as implemented in AMBER11. The same method was applied to study the effect of alanine mutation on mimetic residues R_3_, Y_4_ and R_7_ of **M1**, **M4** and **M6** in complex with IL-10 (S3 Table) [43–45]. Data analysis was carried out with the R-package [65].

In order to ensure that main conclusions inferred from the 10 ns MD simulations maintained valid our strategy design, a further 20 ns extension was carried out for the optimized molecules (*i*.*e*. IL-10/**M2**-**M6** complexes, S6B Fig).

#### GRID calculations

GRID [42, 51] version 22 was used to predict energetically favorable interactions between IL-10 (PDB ID 1J7V) and the following chemical probes: N2 = (sp^2^ amine NH_2_ cation), N3+ (sp^3^ amine NH_3_ cation), COO- (aliphatic carboxylate), and between the unbound IL-10 (PDB ID 2ILK) and the chemical probes: OH2 (water) and C3 (methyl CH_3_ group) (S4 Fig). The GRID box dimensions were set to 51 Å x 58 Å x 46 Å in order to cover the whole protein for the calculations. A grid spacing of 1 Å was applied. The rest of GRID input parameters values were used as default.

#### Structure-based modeling of mIL-10 _C149Y_

The crystal structure of human IL-10 at high resolution (PDB ID 2ILK (1.6 Å)) [27] and its complex structure with IL-10R1 (PDB ID 1J7V (2.9 Å)) [28] were taken as structural templates. Human and mouse proteins and their R1 receptors share high sequence similarities (88% and 73%, respectively). The program Modeller as implemented in Discovery Studio (Accelrys) [66] was used for the modeling. The mouse receptor was modeled following the same procedure.

### Peptide synthesis

Designed IL-10R1 mimetics **M1** to **M4** were purchased from GL Biochem (Shanghai, China) with a purity ≥ 95% by analytical rpHPLC. Their purity was re-checked by rpHPLC and MS before experimental use. Mimetics **M5** and **M6** were synthesized with purity ≥ 95% by analytical rpHPLC using similar methods as previously reported [67].

All commercial reagents and solvents were used without further purification unless otherwise specified. Mass spectra (ESI) were obtained on a Micromass Quattro LC or a Waters Xevo TQ-S mass spectrometer each driven by the Mass Lynx software. Preparative HPLC was performed on a Varian Prepstar system equipped with UV detector (Prostar, Varian) and automatic fraction collector Foxy 200. A Microsorb C18 60–8 column (Varian Dynamax 250 × 21.4 mm) was used as the stationary phase and a binary gradient system of 0.1% TFA/water (solvent A) and 0.1% TFA/CH_3_CN (solvent B) at a flow rate of 10 mL/min served as the eluent. The conditions for the gradient elution are as follows: 0–3 min 90% A, 3–7 min gradient to 30% B, 7–27 min gradient to 65% B, 27–30 min gradient to 95% B, 30–35 min 95% B, 35–40 min gradient back to 90% A. Analytical HPLC was done in a Merck Hitachi system consisting of a L7100 gradient pump combined with a Jasco DG2080 4-line degasser with UV absorption detection at 220 nm by a Merck Hitachi L7450 diode array detector. The system was operated by the D-700 HSM software using a Merck Hitachi D7000 interface. A Luna C18 5 μm column (Phenomenex, 250×4.6 mm) was used as stationary phase and a binary gradient system of 0.1% CF_3_COOH/water (solvent A) and 0.1% TFA/CH_3_CN (solvent B) at a flow rate of 1 mL/min served as the eluent. The programme for elution was as follows: 0–3 min 95% A, 3–25 min gradient to 95% B, 25–30 min 95% B, 30–35 min gradient back to 95% A.

IL-10R1 mimetics **M5** and **M6** were prepared on 0.15 mmol scale by manual stepwise solid phase peptide synthesis using HBTU/DIPEA activation for Fmoc chemistry [68, 69] on Fmoc-Ala-Rink Amide MBHA (substitution 0.37 mmol·g^-1^). Fmoc-Ala-Rink Amide MBHA was prepared by Fmoc-deprotection of Fmoc-Rink Amide MBHA (1 g, substitution 0.55 mmol·g^-1^) resin using the procedure outlined below followed by treating the deprotected Rink Amide MBHA resin with 1.0 equivalent of Fmoc-Ala-OH (0.171 g), 1.25 equivalents of HBTU (0.261 g), and 2.0 equivalents of DIPEA (187 μL) in DMF (4 mL) for 2 hours. Subsequently, the resin was filtered off and washed with DMF and CH_2_Cl_2_ (DCM) (3×5 mL each). Capping of the unreacted resin-bound amino groups was accomplished by treatment with a 1:1 (v/v) mixture of DCM and acetic anhydride. The resin was shaken for 3 min, and the liquid was removed by filtration. The resin was re-suspended in the capping mixture and shaken again for 7 min. After filtration the resin was washed with DCM (3×5 mL), and the presence of remaining resin-bound amino groups was checked using the ninhydrin assay. In the case of positive result, the capping procedure was repeated [70]. Determination of the loading degree was accomplished following the protocol described by Gude *et al*. [71] using two aliquots of the resin. About 5 mg of the resin (exact value has to be noted) containing the Fmoc-Ala-OH was placed into a PP reaction vessel and swollen in DMF (2 mL) for 30 min. The suspension was filtered, and the resin was treated with 2% DBU in DMF (2 mL) and stirred for another 30 min. The suspension was filtered into a graduated 10 mL flask, and the resin was washed with CH_3_CN (3×1 mL). Each filtrate was added to the flask and, the solution was diluted to 10 mL with CH_3_CN. 2 mL of this solution were transferred to a graduated 25 ml flask and diluted with CH_3_CN to 25 mL. A reference solution was prepared the same way but without addition of the resin. The sample solutions were measured against the reference at 294 nm in the UV/Vis spectrometer. The loading yield was calculated as follows: Loading (mmol/g)_294 nm_ = (E×14.214 μmol) / m_resin_(mg). Finally, the average value of the two aliquots was calculated. Using the procedure outlined above, loading degrees in the range of 0.37–0.44 mmol/g were obtained.

Fmoc deprotections were achieved with 2 × 5 min treatments with DMF/Piperidine (1:1). For each coupling, 4.0 equivalents of amino acid and 4.2 equivalents of diisopropylethylamine (DIPEA) were employed in each coupling step (20–25 min), except for Fmoc-Asp(OAll)-OH and Fmoc-Lys(Alloc)-OH, where only 2 equivalents were coupled using HATU and DIPEA activation. Coupling yields were monitored by quantitative ninhydrin assay [72]. After the first Fmoc-Lys(Alloc)-OH residue was coupled, the resin was washed with DMF/DCM, and swollen in DCM. Then, the Ally and Alloc orthogonal protecting groups were removed by treating the peptide resin with PhSiH_3_ in DCM (20 equiv) and Pd(PPh_3_)_4_ (0.15 equiv.) (2 × 20 min) under Ar [73]. After that, the peptide resin was successively washed with DCM, DMF, 0.5% w/v sodium diethyldithiocarbamate in DMF, DMF, and again with DCM. Then, the deprotection step was monitored by using the ninhydrin test, which gave an intense blue, and by MS. Cyclization was effected on-resin using 2.5 equiv. PyBOP, 2.5 equiv. DIPEA in DMF 0.5 M. The reaction was newly monitored by the ninhydrin test and MS after small cleavage with 95% TFA, 2.5% TES, 2.5% H_2_O. The same procedure was repeated to generate the second cycle. The peptide resin was then washed with DMF, MeOH/DCM, DCM, and dried *in vacuo* for 1 hour. Peptide was cleaved by using 95% TFA, 2.5% TES, 2.5% H_2_O (6 mL) for 2 hours. The resin was then filtered off and the solvent evaporated under a stream of nitrogen. The peptide was precipitated with cold diethyl ether. The ether was decanted, and the precipitated peptide was re-dissolved in 1:1 acetonitrile/water and lyophilized. Crude peptide was purified by reverse-phase HPLC and assessed for purity by using the conditions specified above. IL-10R1 mimetic **M5**: *R*_*t*_ 13.0 min, ESMS observed (calculated): [M+2H]^2+^ = 942.18 (942.03), [M+3H]^3+^ = 628.29 (628.36), [M+4H]^4+^ = 471.49 (471.52); IL-10R1 mimetic **M6**: *R*_*t*_ 11.9 min, ESMS observed (calculated): [M+2H]^2+^ = 956.25 (956.04), [M+3H]^3+^ = 637.83 (637.69), [M+4H]^4+^ = 478.67 (478.52).

### Protein expression and purification

Recombinant murine IL-10 (rmIL-10) was expressed in *Escherichia coli* (*E*. *coli*) Rosetta(DE3) (Merck) using a pET41b(+) vector (Novagen). For purification purposes, the C-terminal histidine tag (LEHHHHHHHH) of the vector was used. The rmIL-10 coding region was further modified by replacement of cysteine 149, which is not involved in disulfide bond formation, by a tyrosine, *i*.*e*., the corresponding amino acid in the human IL-10 sequence. That modification improved protein refolding yield by a factor of 20 as previously shown for rat IL-10 but without any loss in activity [48]. The C149Y mutation was introduced according to the instructions of the QuikChange mutagenesis kit (Stratagene). The primers for the introduction of the C149Y mutation were: forward 5’-CTTCATCAACTACATAGAAGC-3’ and reverse complementary 5’-GCTTCTATGTAGTTGATGAAG-3’. Correctness of the rmIL-10 coding region and introduction of the point mutation were confirmed by DNA sequencing.

*E*. *coli* cells were grown at 37°C in a minimal salt medium containing 9.29 g/L sodium sulfate, 29.2 g/L di-potassium hydrogenphosphate, 8.14 g/L sodium dihydrogenphosphate dihydrate, 1.2 g/L magnesium sulfate, 5 g/L glucose, 1 g/L ammonium chloride, 4.92 g/L ammonium sulfate, 100 μg/mL thiamin, 100 μg/mL kanamycin, and 34 μg/mL chloramphenicol. Protein expression was induced with 1 mM isopropyl-*β*-D-thiogalactopyranoside (IPTG) and was conducted for 4 hours. The cell pellet was resuspended in 50 mM Tris-HCl (pH 7.4), 100 mM NaCl, 1 mM EDTA, 0.1 mM PMSF, and cells were lysed by using a french press and by adding 0.3 mg/mL lysozyme. rmIL-10 was obtained in *E*. *coli* inclusion bodies which were washed three times in 50 mM Tris-HCl (pH 7.4), 1 M NaCl, 5% (v/v) Triton X-100, 1 mM EDTA and finally dissolved in 50 M Tris-HCl (pH 7.4), 100 mM NaCl, 6 M guanidine hydrochloride, 200 mM dithiothreitol, 1 mM EDTA. Prior to refolding of rmIL-10, dithiothreitol was removed by dialysis against 100 mM Tris-HCl (pH 9.0), 100 mM NaCl, 6 M guanidine hydrochloride. Protein refolding was initiated by rapid dilution into 100 mM Tris-HCl (pH 9.0), 100 mM NaCl, 1 M L-arginine, 5 mM reduced glutathione, 1 mM oxidized glutathione until a final protein concentration of 0.1 mg/mL was reached. Protein aggregates were immediately removed by filtration of the protein solution through a 0.45 μm nitrocellulose membrane. Refolding was allowed to further proceed for two days.

The rmIL-10 was purified using a Ni-NTA agarose column (Machery-Nagel) equilibrated in 20 mM sodium phosphate (pH 7.4), 50 mM NaCl and was eluted using the same buffer containing 600 mM imidazole. The native rmIL-10 dimer was separated from the monomer and higher molecular aggregates, which form during protein refolding, using a S-200 HR (GE Healthcare) preparative gel filtration column equilibrated in 20 mM sodium phosphate (pH 7.4), 50 mM NaCl. Sample purity and identity were confirmed by SDS-PAGE, N-terminal Edman sequencing and MALDI-TOF mass spectrometry, respectively. Structural integrity of rmIL-10 was confirmed by NMR spectroscopy including the resonance assignments of the protein backbone and Cβ side chain atoms [49].

### Circular Dichroism (CD) and Fluorescence Spectroscopy

CD spectra were recorded with a J-815 instrument (Jasco, Gross-Umstadt, Germany) from 195 to 300 nm in 1-cm cuvettes at concentrations of 2 μM of mIL-10_C149Y_ (dimer) and 4.5 μM of mimetics **M1**-**M4**. Ellipticity θ was recorded in millidegrees. Samples were heated from 298 to 364 K at a rate of 2 K/min under constant stirring to determine stabilization of IL-10/mimetic complexes. Estimates of binding affinities were derived with the assumption of a two state unfolding of mIL-10_C149Y_. The mimetics were assumed to stabilize exclusively the folded state. These simplifications allow applying equilibrium thermodynamics, such that the fraction S_n_ of the native state of mIL-10_C149Y_ can be approximated: S_n_ = (1+ [M] *K*_d_^-1^ +*K*_u_)^-1^. *K*_d_ is the estimated dissociation constant of the mimetic, [M] its concentration. *K*_u_ is the temperature-dependent unfolding equilibrium constant (*K*_u_ = [denatured]/[native]) as measured by the unfolding curve of mIL-10_C149Y_ in the absence of mimetics. This model provides a relative ranking of mimetic affinities but does not allow for a more detailed analysis with respect to absolute binding enthalpy and entropy for which partially unfolded states need to be included. In this case, the measured CD curves would be actually compatible with much higher mimetic affinities, because a local stabilization of the mimetic binding epitope on mIL-10_C149Y_ is not counted as binding in the applied two state model but it is very likely to occur. For consistency, we have not explored the higher affinity limit that would still be compatible with the data. Instead, we report only the lower affinity range that complies with the assumptions, in order to prevent bias for overestimated mimetic affinities. Due to the complicated underlying processes of thermally induced complex dissociation and unfolding of both, mIL-10_C149Y_ and designed mimetics, we cannot relate the shift of the temperature mid point of unfolding (Tm) to more specific thermodynamic parameters by a simple two state unfolding model. Absolute thermodynamic parameters could only be obtained by isothermal titration calorimetry with the most promising mimetics. This of course required the initial confirmation of the binding of the precursor molecules which was accomplished by CD and Fluorescence Spectroscopy. Fluorescence emission at 360 nm was monitored simultaneously with the CD scan (the measuring beam served as the excitation light) using a second monochromator and photomultiplier in 90° geometry with respect to the CD absorption path. Thereby, temperature-dependent excitation spectra of the tryptophan at position two in the synthetic IL-10R1 mimetics were additionally obtained with the CD data acquisition. The thermally induced spectral differences between 334 K and 298 K were calculated for IL-10R1 mimetics in the absence and presence of mIL-10_C149Y_.

### Isothermal titration calorimetry (ITC)

ITC was performed with a Microcal VP-ITC calorimeter (GE Healthcare, Buckinghamshire, U.K.) at 298 K. Aliquots (5–10 μL) of synthetic IL-10R1 mimetics **M5** and **M6** (300–500 mM) dissolved in the original dialysis buffer of the mIL-10_C149Y_ preparation were injected into mIL-10_C149Y_ solutions (19–50 μM) until the molar ratio of mimetic: mIL-10_C149Y_ exceeded 3 and constant enthalpy changes were observed for repeated injections. The final constant heat signals were subtracted to correct for the solution enthalpy of the respective IL-10R1 mimetics. The resulting integrated heat curve was analyzed with the associated software Origin 9 [74] allowing a variable stoichiometry but restriction to a single *K*_d_ value (identical binding sites of the dimeric protein).

## Supporting Information

S1 Fig Cartoon representation of the crystal structure of the complex of human IL-10 (in gray/violet) and IL-10R1 (in brown/yellow) (PDB ID 1J7V, 2.9 Å) and energetic details of IL-10R1 residues involved in IL-10 binding. (A) Two molecules of IL-10R1 bind to two identical two-fold related surface areas of the protein composed of helix A, loop AB and helix F’ of one IL-10 domain. (B) Close-up of the protein-receptor interface. The recognition site involves 27 protein residues (in violet) and 23 receptor residues (in yellow). Selected key binding residues of IL-10R1 (Tyr43, Arg76 and Arg96) and their binding counterparts in IL-10 are shown in sticks, labeled and colored by atom type. The two red spheres represent interfacial crystallographic water molecules interacting with the key binding residues. Intermolecular H-bonds are depicted by black dashed lines. Panels A and B were generated with PyMOL. (C) MM-PBSA IL-10R1 residue binding energy contribution calculated from MD simulation.
(PDF)

S2 Fig Cartoon and surface representation of an IL-10 domain (in gray) in complex with the manually docked selected structural motifs that best matched the defined 3D functional descriptors (Table 1 in main text). Motifs 1ZYL, 2ACA (A), and 2ARZ (B) are represented by a green ribbon, and their helical elongation is depicted in yellow. Those motif residues overlapping with the selected IL-10R1 key binding residues (*i*.*e*. IL-10R1 residues Tyr43, Arg76 and Arg96 in orange) are shown in sticks. The helix axis (represented by a black line) is oriented longitudinally (A) or transversally (B) with respect to the helices A, B, F’ of IL-10. Figure generated with PyMOL.
(PDF)

S3 Fig Lactam bridge design strategy to stabilize the *seeding template* in an alpha-helical scaffold. IL-10 and the scaffolds are represented as gray and green cartoons, respectively. Relevant residues in each of the protein/scaffold complex are labeled and shown in sticks. The scaffolds Ac-[K_1_X_2_R_3_Y_4_D_5_]X_6_R_7_[K_8_X_9_X_10_X_11_D_12_]-NH_2_ (A), Ac-[K_1_X_2_R_3_Y_4_D_5_][K_6_R_7_X_8_X_9_D_10_]X_11_X_12_-NH_2_ (B) and Ac-[K_1_X_2_R_3_Y_4_D_5_][K_6_R_7_K_8_X_9_D_10_]R_11_X_12_-NH_2_ (C) and their respective 180° rotation view are shown. Lactam bridges between the side chains of Lys and Asp are represented by brackets in sequence and in sticks in the structure models. Residue positions available for substitutions in the scaffolds are highlighted with spheres at their C_α_. Figure generated with PyMOL.
(PDF)

S4 Fig Interaction energy maps obtained for IL-10 with a series of GRID chemical probes. (A) Interaction energies calculated for one IL-10 domain from the structure in complex with the receptor-1 (in gray cartoon, PDB ID 1J7V (2.9 Å)) with an N3+ probe (sp^3^ amine NH_3_ cation). Two contour energy levels are shown at cutoffs -7.5 kcal/mol and -13.5 kcal/mol (cyan and blue, respectively). The most favorable interactions with IL-10 involve residues from helix A (Gln38) and loop AB (Asp41, Gln42) (highlighted in pink). Further favorable interactions in the same region comprise residues of the loop AB (Asp44) and helix F’ (Ser141, Glu142, Asp144) (highlighted in violet). (B) Interaction energies calculated for the unbound IL-10 (PDB ID 2ILK (1.6 Å)) with a water molecule or “*solvent*” chemical probe (contour energy level at cutoff -7.5 kcal/mol shown in red) and for a carbon sp^3^ or “*solvent exclusion*” chemical probe (contour energy level at cutoff -2.8 kcal/mol shown in green). Favorable interactions of both, *solvent* and *solvent exclusion* probes, overlap with the mimetic binding region indicating solvent exclusion. Three crystallographic waters found in the free IL-10 in this region are shown as yellow spheres. Figure generated in VMD.
(PDF)

S5 Fig Molecular modeling of murine IL-10 mutant mIL-10_C149Y_. Top: sequence alignment of murine and human proteins used for the homology modeling (sequence numbering as in PDB ID 2ILK). Bottom: detail of one domain of the obtained 3D molecular model of mIL-10_C149Y_ (beige cartoon) superimposed with one domain of the human IL-10 used as template (PDB ID 2ILK, gray cartoon) (RMSD_Cα_: 0.8 Å). The protein residues involved in mimetic recognition are shown in sticks (pink for murine and violet for human. Figure generated with PyMOL.
(PDF)

S6 Fig Surface representation of a domain of IL-10 (in gray) in complex with mimetic M5 (in green, N-terminal functionalization in yellow) and RMSD along MD simulation. (A) Hydrophobic interactions between mimetic residues K_8_, Y_4_, K_-1_ and protein residues D44, L46 and I145 are highlighted with labels. Structure taken from a snapshot of the MD simulation. Figure generated with PyMOL. (B) Relative movement of mimetic M5 (given as backbone RMSD) with respect to IL-10 along a 30 ns MD simulation.
(PDF)

S1 Table List of motifs in the PDB database that match the *3D pattern syntax* query R-<2,6>-R-<4,5>-Y-. Matches corresponding to short regular secondary structures (maximum 10 residues long in the same chain) are highlighted in bold.
(PDF)

S2 Table Summary of dynamic H-bond formation between IL-10 and IL-10R1 mimetics M1-M6 along MD simulations. M1: Ac-[KW_2_R_3_Y_4_D][KR_7_K_8_VD]R_11_A-NH_2_, M2: X-[KWR_3_Y_4_D][KR_7_K_8_VD]R_11_A-NH_2_ (X = 4-guanidinobutanoyl), M3: X-[KZR_3_Y_4_D][KR_7_K_8_VD]R_11_A-NH_2_ (X = 4-guanidinobutanoyl, Z = 5-Hydroxy-L-tryptophan), M4: Ac-R_-1_[KWR_3_Y_4_D][KR_7_K_8_VD]R_11_A-NH_2_, M5: Ac-E_-2_K_-1_[KWR_3_Y_4_D][KR_7_K_8_VD]R_11_A-NH_2_, M6: Ac-E_-2_R_-1_[KWR_3_Y_4_D][KR_7_K_8_VD]R_11_A-NH_2_. ^#^H-bonds observed along MD simulations of 10 ns for IL-10/M1 and 30 ns for IL-10/M2-M6 are shown in black. Additional H-bonds observed during last 10 ns MD production for IL-10/M2-M6 are shown in gray. *Interactions via structural bridging water. S: side chain. M: main chain. ^a^Not observed during the last 10 ns MD production.
(PDF)

S3 Table Binding free energies for alanine mutants of M1, M4 and M6 calculated with the MM-PBSA alanine scanning method. (PDF)
