# Supplementary material for: Rational Structure-Based Rescaffolding Approach to De Novo Design of Interleukin 10 (IL-10) Receptor-1 Mimetics
Source: PLoS One. 2016 Apr 28;11(4):e0154046. doi: 10.1371/journal.pone.0154046 (PMC4849758; doi:10.1371/journal.pone.0154046)
Supplement: S1 Fig — (A) Two molecules of IL-10R1 bind to two identical two-fold related surface areas of the protein composed of helix A, loop AB and helix F’ of one IL-10 domain. (B) Close-up of the protein-receptor interface. The recognition site involves 27 protein residues (in violet) and 23 receptor residues (in yellow). Selected key binding residues of IL-10R1 (Tyr43, Arg76 and Arg96) and their binding counterparts in IL-10 are shown in sticks, labeled and colored by atom type. The two red spheres represent interfacial crystallographic water molecules interacting with the key binding residues. Intermolecular H-bonds are depicted by black dashed lines. Panels A and B were generated with PyMOL. (C) MM-PBSA IL-10R1 residue binding energy contribution calculated from MD simulation. (PDF) [file pone.0154046.s001.pdf]

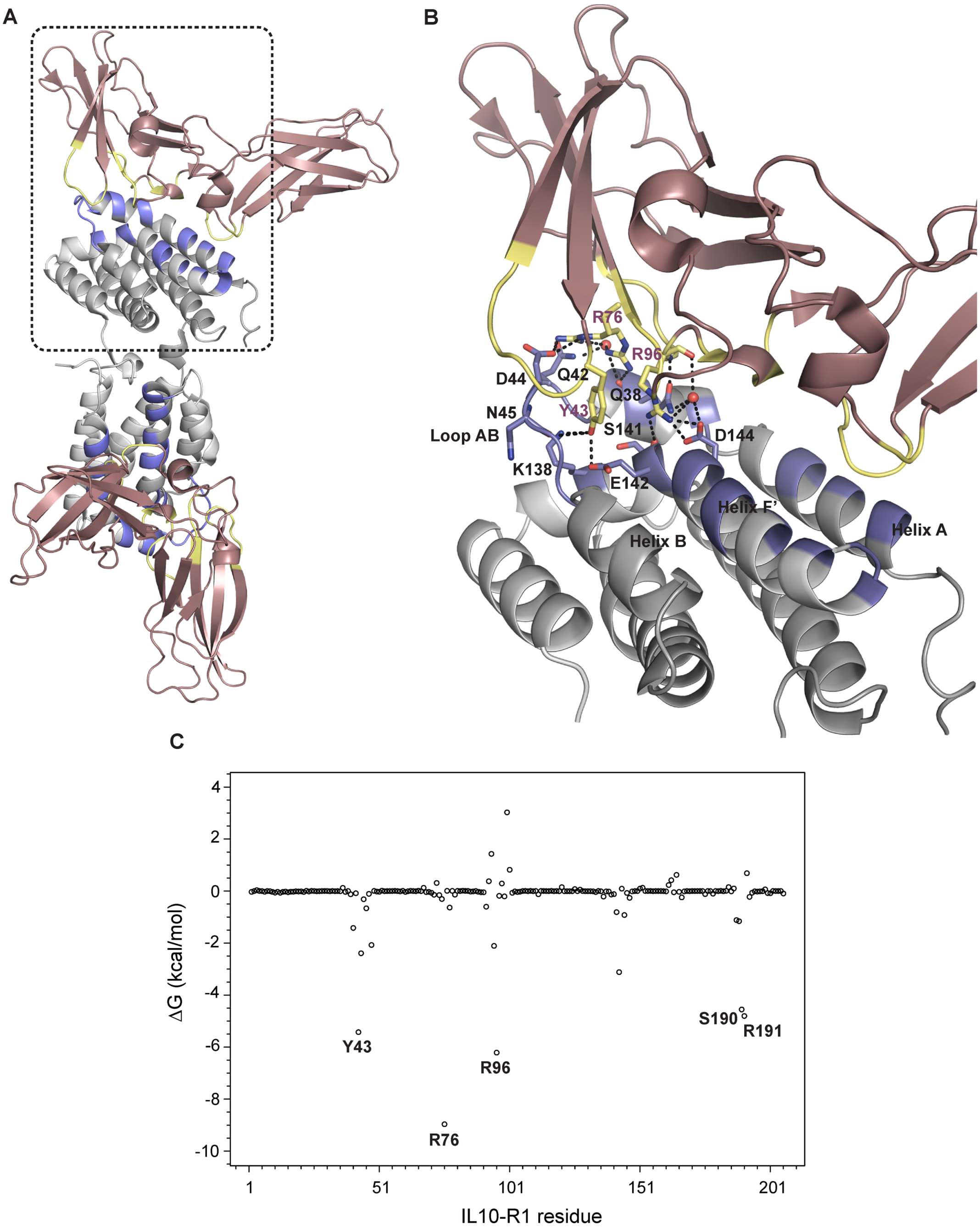

S1 Fig. Cartoon representation of the crystal structure of the complex of human IL-10 (in gray/violet) and IL-10R1 (in brown/yellow) (PDB ID 1J7V, 2.9 Å) and energetic details of IL-10R1 residues involved in IL-10 binding. (A) Two molecules of IL-10R1 bind to two identical two-fold related surface areas of the protein composed of helix A, loop AB and helix F' of one IL-10 domain. (B) Close-up of the protein-receptor interface. The recognition site involves 27 protein residues (in violet) and 23 receptor residues (in yellow). Selected key binding residues of IL-10R1 (Tyr43, Arg76 and Arg96) and their binding counterparts in IL-10 are shown in sticks, labeled and colored by atom type. The two red spheres represent interfacial crystallographic water molecules interacting with the key binding residues. Intermolecular H-bonds are depicted by black dashed lines. Panels A and B were generated with PyMOL. (C) MM-PBSA IL-10R1 residue binding energy contribution calculated from MD simulation.
