# Supplementary material for: Rational Structure-Based Rescaffolding Approach to De Novo Design of Interleukin 10 (IL-10) Receptor-1 Mimetics
Source: PLoS One. 2016 Apr 28;11(4):e0154046. doi: 10.1371/journal.pone.0154046 (PMC4849758; doi:10.1371/journal.pone.0154046)
Supplement: S2 Fig — Motifs 1ZYL, 2ACA (A), and 2ARZ (B) are represented by a green ribbon, and their helical elongation is depicted in yellow. Those motif residues overlapping with the selected IL-10R1 key binding residues (i.e. IL-10R1 residues Tyr43, Arg76 and Arg96 in orange) are shown in sticks. The helix axis (represented by a black line) is oriented longitudinally (A) or transversally (B) with respect to the helices A, B, F’ of IL-10. Figure generated with PyMOL. (PDF) [file pone.0154046.s002.pdf]

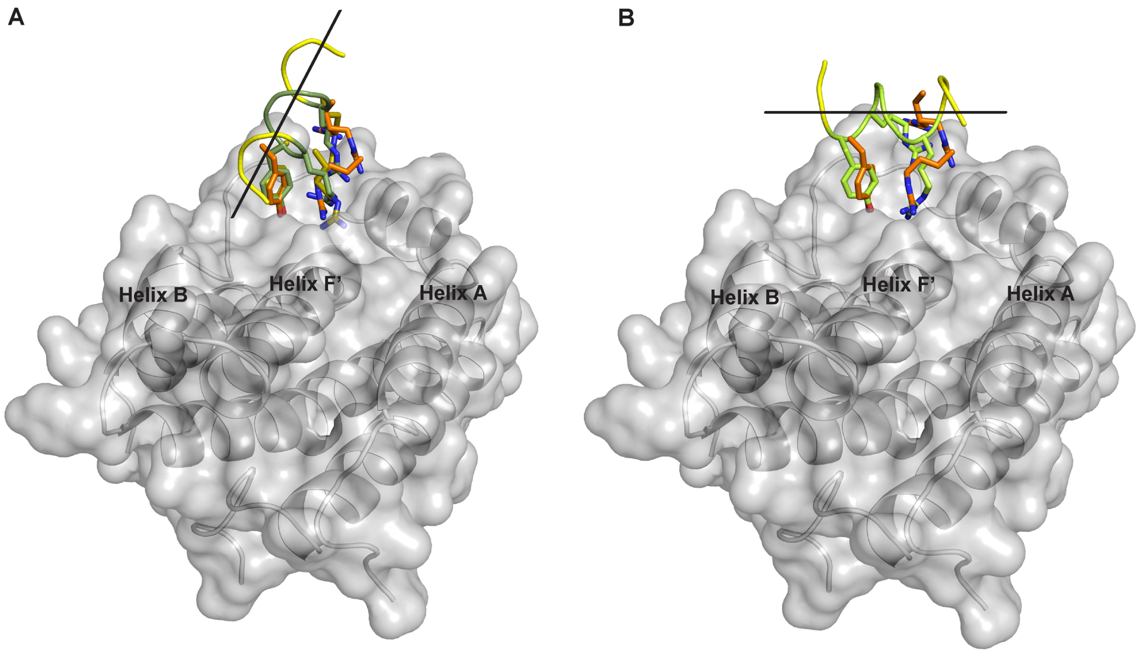

S2 Fig. Cartoon and surface representation of an IL-10 domain (in gray) in complex with the manually docked selected structural motifs that best matched the defined 3D functional descriptors (Table 1 in main text). Motifs 1ZYL, 2ACA (A), and 2ARZ (B) are represented by a green ribbon, and their helical elongation is depicted in yellow. Those motif residues overlapping with the selected IL-10R1 key binding residues (*i.e.* IL-10R1 residues Tyr43, Arg76 and Arg96 in orange) are shown in sticks. The helix axis (represented by a black line) is oriented longitudinally (A) or transversally (B) with respect to the helices A, B, F' of IL-10. Figure generated with PyMOL.
