# Supplementary material for: Rational Structure-Based Rescaffolding Approach to De Novo Design of Interleukin 10 (IL-10) Receptor-1 Mimetics
Source: PLoS One. 2016 Apr 28;11(4):e0154046. doi: 10.1371/journal.pone.0154046 (PMC4849758; doi:10.1371/journal.pone.0154046)
Supplement: S3 Fig — IL-10 and the scaffolds are represented as gray and green cartoons, respectively. Relevant residues in each of the protein/scaffold complex are labeled and shown in sticks. The scaffolds Ac-[K1X2R3Y4D5]X6R7[K8X9X10X11D12]-NH2 (A), Ac-[K1X2R3Y4D5][K6R7X8X9D10]X11X12-NH2 (B) and Ac-[K1X2R3Y4D5][K6R7K8X9D10]R11X12-NH2 (C) and their respective 180° rotation view are shown. Lactam bridges between the side chains of Lys and Asp are represented by brackets in sequence and in sticks in the structure models. Residue positions available for substitutions in the scaffolds are highlighted with spheres at their Cα. Figure generated with PyMOL. (PDF) [file pone.0154046.s003.pdf]

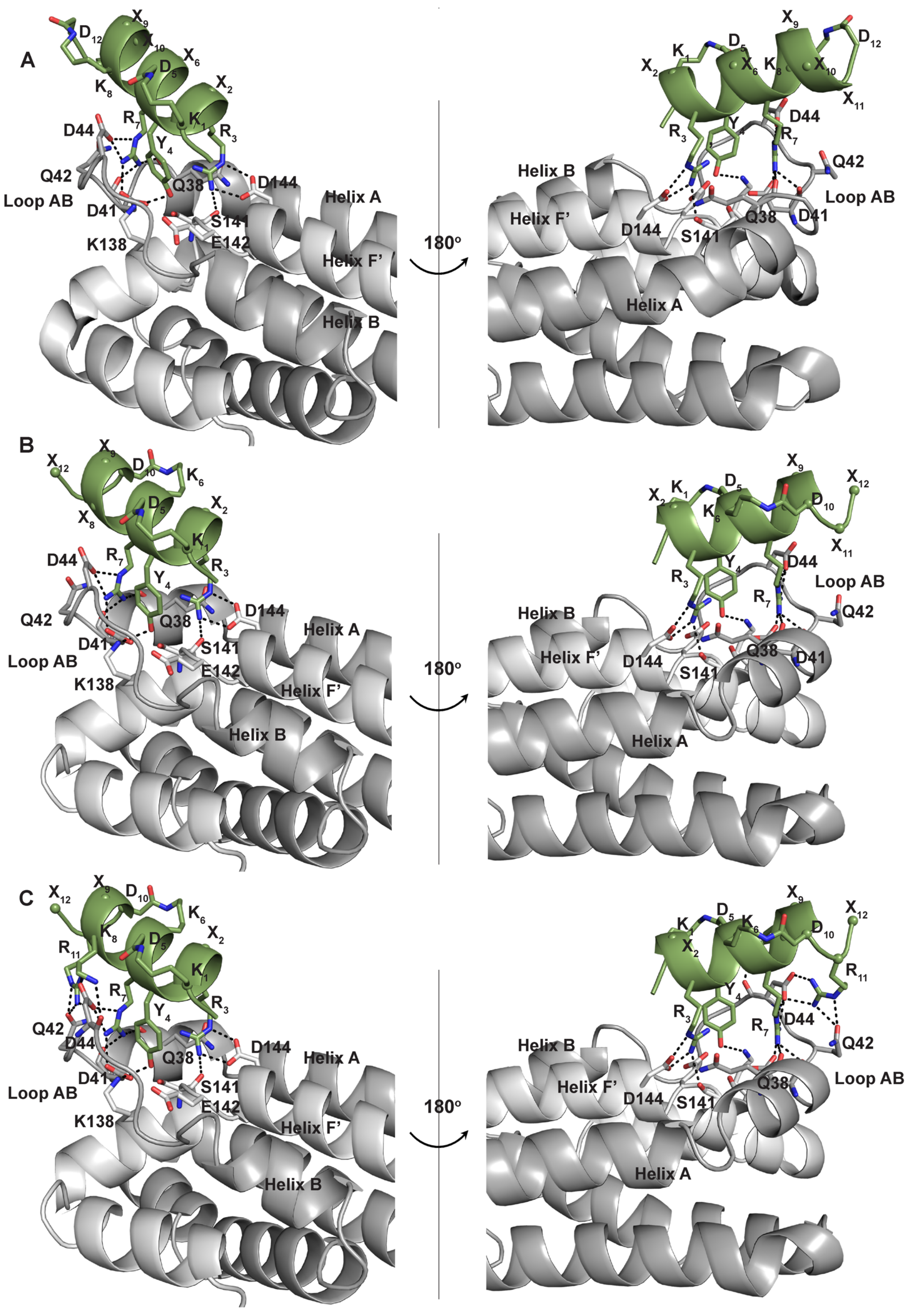

S3 Fig. Lactam bridge design strategy to stabilize the *seeding template* in an alpha-helical scaffold. IL-10 and the scaffolds are represented as gray and green cartoons, respectively. Relevant residues in each of the protein/scaffold complex are labeled and shown in sticks. The scaffolds Ac-[$K_1X_2R_3Y_4D_5$]X_6R_7[$K_8X_9X_{10}X_{11}D_{12}$]-NH_2 (A), Ac-[$K_1X_2R_3Y_4D_5$][$K_6R_7X_8X_9D_{10}$]$X_{11}X_{12}$-NH_2 (B) and Ac-[$K_1X_2R_3Y_4D_5$][$K_6R_7K_8X_9D_{10}$]$R_{11}X_{12}$-NH_2 (C) and their respective 180° rotation view are shown. Lactam bridges between the side chains of Lys and Asp are represented by brackets in sequence and in sticks in the structure models. Residue positions available for substitutions in the scaffolds are highlighted with spheres at their C_α. Figure generated with PyMOL.
