# Supplementary material for: Rational Structure-Based Rescaffolding Approach to De Novo Design of Interleukin 10 (IL-10) Receptor-1 Mimetics
Source: PLoS One. 2016 Apr 28;11(4):e0154046. doi: 10.1371/journal.pone.0154046 (PMC4849758; doi:10.1371/journal.pone.0154046)
Supplement: S4 Fig — (A) Interaction energies calculated for one IL-10 domain from the structure in complex with the receptor-1 (in gray cartoon, PDB ID 1J7V (2.9 Å)) with an N3+ probe (sp3 amine NH3 cation). Two contour energy levels are shown at cutoffs -7.5 kcal/mol and -13.5 kcal/mol (cyan and blue, respectively). The most favorable interactions with IL-10 involve residues from helix A (Gln38) and loop AB (Asp41, Gln42) (highlighted in pink). Further favorable interactions in the same region comprise residues of the loop AB (Asp44) and helix F’ (Ser141, Glu142, Asp144) (highlighted in violet). (B) Interaction energies calculated for the unbound IL-10 (PDB ID 2ILK (1.6 Å)) with a water molecule or “solvent” chemical probe (contour energy level at cutoff -7.5 kcal/mol shown in red) and for a carbon sp3 or “solvent exclusion” chemical probe (contour energy level at cutoff -2.8 kcal/mol shown in green). Favorable interactions of both, solvent and solvent exclusion probes, overlap with the mimetic binding region indicating solvent exclusion. Three crystallographic waters found in the free IL-10 in this region are shown as yellow spheres. Figure generated in VMD. (PDF) [file pone.0154046.s004.pdf]

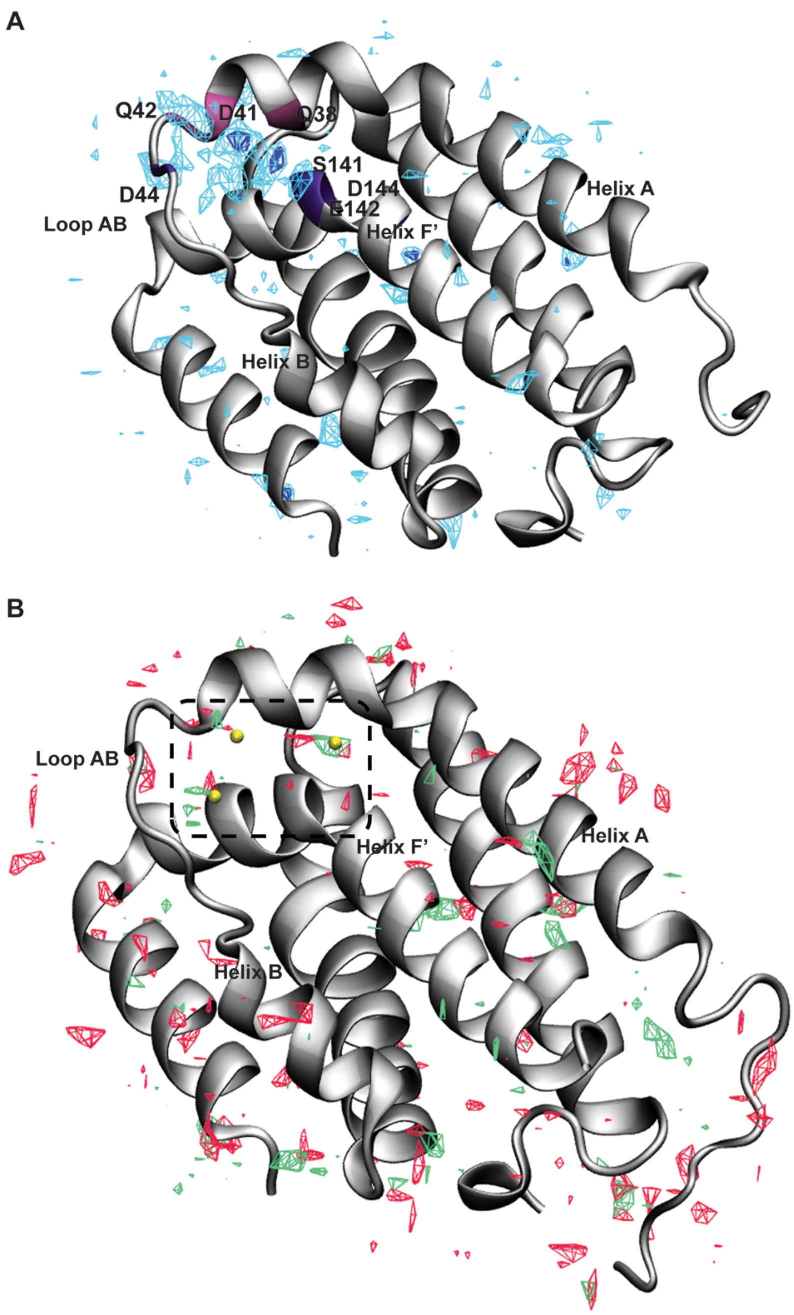


**S4 Fig. Interaction energy maps obtained for IL-10 with a series of GRID chemical probes.** (A) Interaction energies calculated for one IL-10 domain from the structure in complex with the receptor-1 (in gray cartoon, PDB ID 1J7V (2.9 Å)) with an N3+ probe ($sp^3$ amine $NH_3$ cation). Two contour energy levels are shown at cutoffs -7.5 kcal/mol and -13.5 kcal/mol (cyan and blue, respectively). The most favorable interactions with IL-10 involve residues from helix A (Gln38) and loop AB (Asp41, Gln42) (highlighted in pink). Further favorable interactions in the same region comprise residues of the loop AB (Asp44) and helix F' (Ser141, Glu142, Asp144) (highlighted in violet). (B) Interaction energies calculated for the unbound IL-10 (PDB ID 2ILK (1.6 Å)) with a water molecule or "*solvent*" chemical probe (contour energy level at cutoff -7.5 kcal/mol shown in red) and for a carbon $sp^3$ or "*solvent exclusion*" chemical probe (contour energy level at cutoff -2.8 kcal/mol shown in green). Favorable interactions of both, *solvent* and *solvent exclusion* probes, overlap with the mimetic binding region indicating solvent exclusion. Three crystallographic waters found in the free IL-10 in this region are shown as yellow spheres. Figure generated in VMD.
