# Supplementary material for: Rational Structure-Based Rescaffolding Approach to De Novo Design of Interleukin 10 (IL-10) Receptor-1 Mimetics
Source: PLoS One. 2016 Apr 28;11(4):e0154046. doi: 10.1371/journal.pone.0154046 (PMC4849758; doi:10.1371/journal.pone.0154046)
Supplement: S5 Fig — Top: sequence alignment of murine and human proteins used for the homology modeling (sequence numbering as in PDB ID 2ILK). Bottom: detail of one domain of the obtained 3D molecular model of mIL-10C149Y (beige cartoon) superimposed with one domain of the human IL-10 used as template (PDB ID 2ILK, gray cartoon) (RMSDCα: 0.8 Å). The protein residues involved in mimetic recognition are shown in sticks (pink for murine and violet for human. Figure generated with PyMOL. (PDF) [file pone.0154046.s005.pdf]

```
                         12         22         32         42
murine IL-10C149Y     SREDNNC THFPVGQSHM LLELRTAFSQ VKTFFQTKDQ
human IL-10           TQSENSC THFPGNLPNM LRDLRDAFSR VKTFFQMKDQ

                      52         62         72         82
murine IL-10C149Y  LDNILLTDSL MQDFKGYLGC QALSEMIQFY LVEVMPQAEK
human IL-10        LDNLLLKESL LEDFKGYLGC QALSEMIQFY LEEVMPQAEN

                      92        102        112        122
murine IL-10C149Y  HGPEIKEHLN SLGEKLKTLR MRLRRCHRFL PCENKSKAVE
human IL-10        QDPDIKAHVN SLGENLKTLR LRLRRCHRFL PCENKSKAVE

                     132        142        152      160
murine IL-10C149Y  QVKSDFNKLQ DQGVYKAMNE FDIFINYIEA YMMIKMKS
human IL-10        QVKNAFNKLQ EKGIYKAMSE FDIFINYIEA YMTMKIRN
```

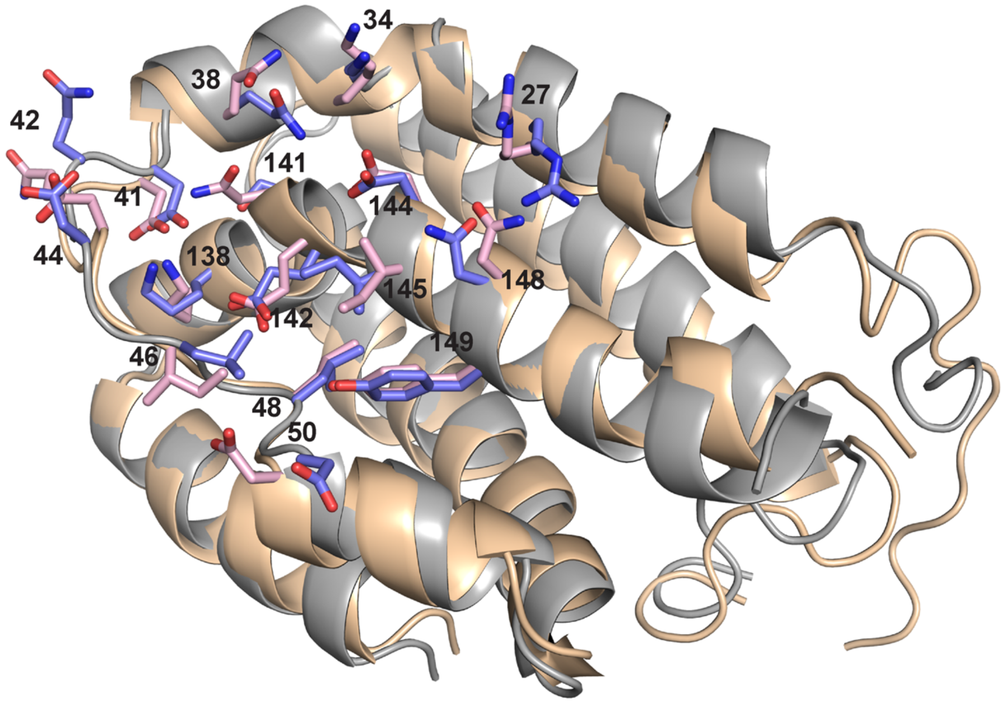

S5 Fig. Molecular modeling of murine IL-10 mutant mIL-10$_{C149Y}$. Top: sequence alignment of murine and human proteins used for the homology modeling (sequence numbering as in PDB ID 2ILK). Bottom: detail of one domain of the obtained 3D molecular model of mIL-10$_{C149Y}$ (beige cartoon) superimposed with one domain of the human IL-10 used as template (PDB ID 2ILK, gray cartoon) (RMSD$_{C\alpha}$: 0.8 Å). The protein residues involved in mimetic recognition are shown in sticks (pink for murine and violet for human. Figure generated with PyMOL.
