# Supplementary material for: Rational Structure-Based Rescaffolding Approach to De Novo Design of Interleukin 10 (IL-10) Receptor-1 Mimetics
Source: PLoS One. 2016 Apr 28;11(4):e0154046. doi: 10.1371/journal.pone.0154046 (PMC4849758; doi:10.1371/journal.pone.0154046)
Supplement: S6 Fig — (A) Hydrophobic interactions between mimetic residues K8, Y4, K-1 and protein residues D44, L46 and I145 are highlighted with labels. Structure taken from a snapshot of the MD simulation. Figure generated with PyMOL. (B) Relative movement of mimetic M5 (given as backbone RMSD) with respect to IL-10 along a 30 ns MD simulation. (PDF) [file pone.0154046.s006.pdf]

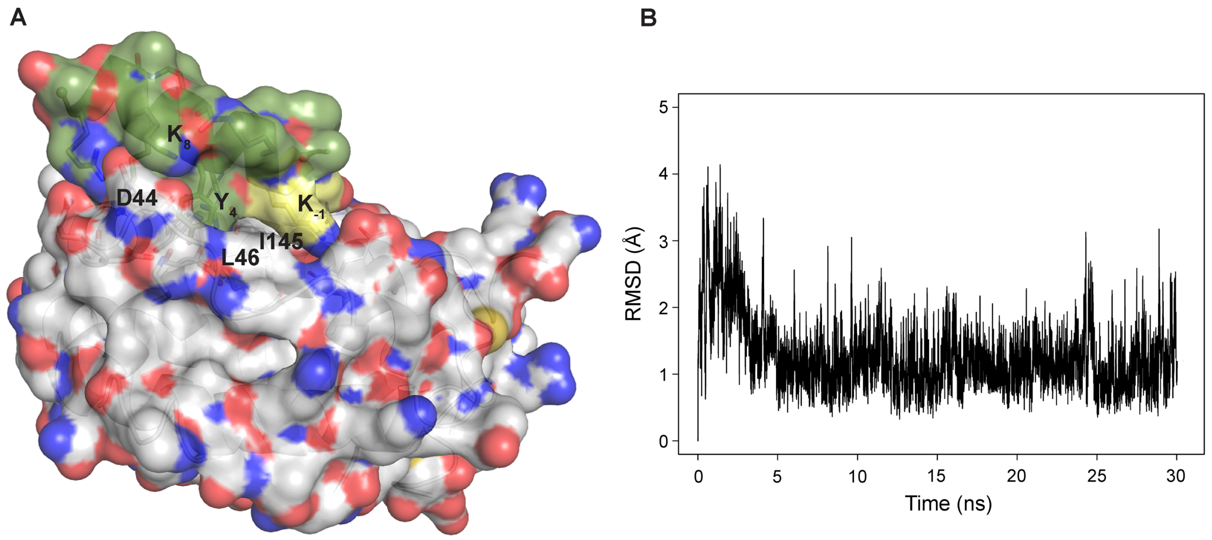

S6 Fig. Surface representation of a domain of IL-10 (in gray) in complex with mimetic M5 (in green, N-terminal functionalization in yellow) and RMSD along MD simulation. (A) Hydrophobic interactions between mimetic residues K_8, Y_4, K_{-1} and protein residues D44, L46 and I145 are highlighted with labels. Structure taken from a snapshot of the MD simulation. Figure generated with PyMOL. (B) Relative movement of mimetic **M5** (given as backbone RMSD) with respect to IL-10 along a 30 ns MD simulation.
