# Supplementary material for: Rational Structure-Based Rescaffolding Approach to De Novo Design of Interleukin 10 (IL-10) Receptor-1 Mimetics
Source: PLoS One. 2016 Apr 28;11(4):e0154046. doi: 10.1371/journal.pone.0154046 (PMC4849758; doi:10.1371/journal.pone.0154046)
Supplement: S1 Table — Matches corresponding to short regular secondary structures (maximum 10 residues long in the same chain) are highlighted in bold. (PDF) [file pone.0154046.s007.pdf]

S1 Table. List of motifs in the PDB database that match the *3D pattern syntax* query R-<2,6>-R-<4,5>-Y-. Matches corresponding to short regular secondary structures (maximum 10 residues long in the same chain) are highlighted in bold.

Sequence: sequence of fragments containing matching residues (in bold)
Structure: secondary structure of fragments containing matching residues
Dots represent residues in the sequence when this is longer than 10

PDB ID	Matching Residues	PDB Chain	Sequence and structure details of hits
2AO9	R_{118}<br>R_{91}<br>Y_{95}	A	$_{91}$-**R**EQV**Y**SKLMQ...QLYMQ**R**-$_{118}$<br>αααααααααα... αααααα
**2ARZ**	R_{108}<br>R_{105}<br>Y_{109}	A	$_{105}$-**R**YY**RY**-$_{109}$<br>ααααα
3BE3	R_{9}<br>R_{9}<br>Y_{74}	A<br>B	$_{9}$-**R**PGVYR<br>---βββ<br>$_{9}$-**R**PGVYR...QPRFA**Y**-$_{74}$<br>---βββ...ββ-βββ
3BF4	R_{108}<br>R_{105}<br>Y_{39}	A<br>B	$_{105}$-**R**SD**R**-$_{108}$<br>----<br>$_{39}$-**Y**YTVEK<br>ββββββ
3D19	R_{22}<br>R_{135}<br>Y_{131}	A	$_{22}$-**R**SLNEI...SREAD**Y**FRK**R**-$_{135}$<br>αααααα ... αααααααααα
3DME	R_{21}<br>R_{81}<br>Y_{77}	A	$_{21}$-**R**ALAAG...HLLYE**Y**CAA**R**-$_{81}$<br>αααααα ... αααααααααα
3DNU	R_{428}<br>R_{432}<br>Y_{370}	A	$_{370}$-**Y**PRHFL...ESNVL**R**LHG**R**-$_{432}$<br>--αααα...αααααααααα
2DJW	R_{68}<br>R_{9}<br>Y_{38}	A<br>B	$_{68}$-**R**TETLL<br>ββββββ<br>$_{9}$-**R**PRGNR...SVTGP**Y**-$_{38}$<br>β------...β-----
3E48	R_{139}<br>R_{194}<br>Y_{137}	B	$_{137}$-**Y**V**R**M^{SE}AM^{SE}YM^{SE}...DTWGK**R**-$_{194}$<br>ββββ -- -- ...-----β
3E49	R_{187}<br>R_{143}<br>Y_{121}	D<br>C	$_{187}$-**R**KLATP<br>α-----<br>$_{121}$-**Y**PM^{SE}LER...HLERS**R**-$_{143}$<br>αα α ααα...ααααα-
3EGL	R_{215}<br>R_{92}<br>Y_{124}	B<br>A	$_{215}$-**R**EAAK^{M}QL<br>ααααααα<br>$_{92}$-**R**VVDTS...SLQEC**Y**-$_{124}$<br>ββ----...αααααα
2DST	R_{3}<br>R_{18}<br>Y_{46}	A	$_{3}$-**R**AGYLH...NLVFD**R**VGKGPP...EGYAF**Y**-$_{46}$<br>ββββββ...βββββββ------...---βββ
3ER6	R_{19}<br>R_{58}<br>Y_{20}	A	$_{19}$-**RY**FASII...RPLIG**R**-$_{58}$<br>--ααααα...--ββ--

2ESN	R_{61}<br>R_{23}<br>Y_{20}	C	$_{20}$-**Y**RH**R**NVGTA...LRQGN**R**-$_{61}$<br>αα----ααα...βββ--β
3EO4	R_{468}<br>R_{492}<br>Y_{379}	A	$_{379}$-**Y**IQKEP...KKAHA**R**ILENN...KTKKG**R**-$_{492}$<br>------...-ββββββ----...βββ---
2EPG	R_{426}<br>R_{469}<br>Y_{379}	B	$_{379}$-**Y**SYVLA...KELAE**R**GILVR...KVARL**R**-$_{469}$<br>-βββββ...αααααα--βββ...ββββββ
2F4I	R_{118}<br>R_{115}<br>Y_{202}	B	$_{115}$-**R**ENR**K**^{M}VPDL...DEEIK^{M}**Y**-$_{202}$<br>----- ---β...------ -
2FEF	R_{59}<br>R_{54}<br>Y_{251}	A<br>C	$_{54}$-**R**SHGY**R**-$_{59}$<br>α---αα<br>$_{251}$-**Y**AWGWW<br>------
3FGB	R_{236}<br>R_{331}<br>Y_{43}	A	$_{43}$-**Y**TSGDS...AGSGP**R**HLTFA...TGIHP**R**-$_{331}$<br>------... -----ββββββ...------
3DZM	R_{135}<br>R_{36}<br>Y_{97}	A	$_{36}$-**R**LGVGFA...VEVAP**Y**FGVRY...LGLGV**R**-$_{135}$<br>ββββββ...-ββββββββββ...ββββββ
2FZF	R_{141}<br>R_{155}<br>Y_{151}	A<br>B	$_{141}$-**R**YLDAM<br>αααααα<br>$_{151}$-**Y**YLT**R**-$_{155}$<br>ααααα
3G7G	R_{95}<br>R_{145}<br>Y_{116}	A	$_{95}$-**R**TVPDE...VDPNA**Y**YFRTI...VCCAS**R**-$_{145}$<br>ββ--αα...-ααααα-ββββ...βββββ-
2GSC	R_{39}<br>R_{47}<br>Y_{28}	A	$_{28}$-**Y**RLTEVFPDQE**R**YGLTAQL**R**-$_{47}$<br>αααααα------ααααααα
3FD9	R_{143}<br>R_{58}<br>Y_{128}	A<br>B	$_{128}$-**Y**GWWES...AIPGW**R**-$_{143}$<br>αααααα... αααααα<br>$_{58}$-**R**M^{SE}LPR<br>α - ---
3GJU	R_{151}<br>R_{190}<br>Y_{187}	A	$_{151}$-**R**GYHGS...HTEAP**Y**YF**R**-$_{190}$<br>------...------ααα
3GMI	R_{274}<br>R_{331}<br>Y_{282}	A	$_{274}$-**R**ILETANE**Y**DLYEY...SKLEA**R**-$_{331}$<br>αααααααα---ααα...ααααα-
3GMS	R_{267}<br>R_{57}<br>Y_{54}	A	$_{54}$-**Y**AH**R**IPLPN...NIFHL**R**-$_{267}$<br>---------...----αα
3GN3	R_{48}<br>R_{50}<br>Y_{143}	A	$_{48}$-**R**I**R**LQSQP...KWHTK**Y**-$_{143}$<br>ββββ----...αααααα
2HNE	R_{100}<br>R_{151}<br>Y_{152}	A	$_{100}$-**R**WLGPE...DTIDF**RY**-$_{152}$<br>ααα---...α------
2HEK	R_{162}<br>R_{213}<br>Y_{217}	A	$_{162}$-**R**MDYLR...NFLIS**R**YFM**Y**-$_{217}$<br>αααααα...αααααααααα

2HXT	R_{100}<br>R_{151}<br>Y_{152}	A	$_{100}$-**R**WLGPE...DTIDF**RY**-$_{152}$<br>ααα---...α------
2HXU	R_{100}<br>R_{151}<br>Y_{152}	A	$_{100}$-**R**WLGPE...DTIDF**RY**-$_{152}$<br>ααα---...α------
1IHN	R_{43}<br>R_{25}<br>Y_{27}	B<br><br>A	$_{43}$-**R**KEISR<br>-ααααα<br>$_{25}$-**R**E**Y**-$_{27}$<br>βββ
2IM9	R_{145}<br>R_{156}<br>Y_{153}	A	$_{145}$-**R**YKNGKRS**Y**IN**R**-$_{156}$<br>α-----------
2HQY	R_{276}<br>R_{118}<br>Y_{123}	A	$_{118}$-**R**AYAD**Y**IYLRS...GIEGL**R**-$_{276}$-$_{123}$<br>----ββββ-αα...--αααα
2HV2	R_{193}<br>R_{36}<br>Y_{189}	F	$_{36}$-**R**FEKLL...TWWLD**Y**TLN**R**-$_{193}$<br>αααααα...ααααααααα-
2I6H	R_{73}<br>R_{160}<br>Y_{83}	A	$_{73}$-**R**NCFRGTAHM^{SE}**Y**ATDPL...GRFPH**R**-$_{160}$<br>αααα---αα α α-αααα... ---ααα
2IDG	R_{126}<br>R_{78}<br>Y_{79}	A	$_{78}$-**RY**GFQAS...RAAQH**R**-$_{126}$<br>αα-----... αααααα
2IT2	R_{148}<br>R_{148}<br>Y_{132}	A<br><br>B	$_{132}$-**Y**SNIKSILIVEI**R**-$_{148}$<br>--βββ----ββββ<br>$_{148}$-**R**STERM^{SE}<br>β-----
2IT3	R_{148}<br>R_{148}<br>Y_{132}	B<br><br>A	$_{148}$-**R**STERM^{SE}<br>β-----<br>$_{132}$-**Y**SNIKSILIVEI**R**-$_{148}$<br>--βββ----ββββ
2JYN	R_{133}<br>R_{106}<br>Y_{30}	A	$_{30}$-**Y**WKLLT...FGTLL**R**TDASA...AFEIA**R**-$_{145}$<br>αααααα...--ββββ-----...αααααα
3FDJ	R_{224}<br>R_{120}<br>Y_{253}	A	$_{120}$-**R**IILEQ...EGGKL**R**ICHVE...TDVCV**Y**-$_{253}$<br>αααααα...-----βββββ--...--ββββ
1MW7	R_{203}<br>R_{133}<br>Y_{179}	A	$_{133}$-**R**KSVFE...IIRGD**Y**NSFKL...KASLQ**R**-$_{203}$<br>ββββββ...ββββ----ααα...ββββββ
2NYG	R_{120}<br>R_{127}<br>Y_{101}	A	$_{101}$-**Y**NSNY...IVELF**R**SYPEVK**R**-$_{127}$<br>------...αααααα-------
1O1Y	R_{146}<br>R_{147}<br>Y_{164}	A	$_{146}$-**RR**ATRVF...NQGFV**Y**-$_{164}$<br>---ββββ...--βββ-
2P3Y	R_{295}<br>R_{405}<br>Y_{343}	B	$_{295}$-**R**AM^{SE}AQS...RAAWF**Y**EAITV...YDENN**R**-$_{405}$<br>αα α ααα...ααααααα----...β-----
2PH7	R_{112}<br>R_{127}<br>Y_{128}	B	$_{112}$-**R**EVTDR...YEAEG**RY**-$_{128}$<br>αααααα...-βββ---

1P8C	R_{100}<br>R_{10}<br>Y_{65}	B<br>E	$_{100}$-**R**RAVGF<br>αααααα<br>$_{10}$-**R**RELNE...DDCIR**Y**-$_{65}$<br>αααααα...αααααα
2P90	R_{70}<br>R_{73}<br>Y_{35}	A<br>B	$_{70}$-**R**SR**R**-$_{73}$<br>αααα<br>$_{35}$-**Y**ADAGH<br>------
2P9J	R_{61}<br>R_{69}<br>Y_{27}	A<br>B	$_{27}$-**Y**TEHGE...AVISG**R**-$_{61}$<br>β----β...βββ---<br>$_{69}$-**R**LLELG<br>αααααα
**2G2X**	R_{39}<br>R_{35}<br>Y_{32}	A	$_{32}$-**Y**QA**R**NFL**R**-$_{39}$<br>αααααααα
2QE8	R_{220}<br>R_{278}<br>Y_{243}	A	$_{220}$-**R**IKSAD...SKIER**Y**SEKPI...ITSAD**R**-$_{278}$<br>ββ-ααα...αα-βββββ---...------
2QJV	R_{201}<br>R_{207}<br>Y_{216}	A<br>B	$_{201}$-**R**VYTDD**R**-$_{207}$<br>βββ----<br>$_{216}$-**Y**NRDVV<br>β---ββ
1PT5	R_{217}<br>R_{213}<br>Y_{59}	A<br>B	$_{213}$-**R**DQQ**R**-$_{217}$<br>ααααα<br>$_{59}$-**Y**FTLMN<br>αααααα
1PT7	R_{217}<br>R_{213}<br>Y_{59}	A<br>B	$_{213}$-**R**DQQ**R**-$_{217}$<br>ααααα<br>$_{59}$-**Y**FTLMN<br>αααααα
1PT8	R_{217}<br>R_{213}<br>Y_{59}	A<br>B	$_{213}$-**R**DQQ**R**-$_{217}$<br>ααααα<br>$_{59}$-**Y**FTLMN<br>αααααα
2R6S	R_{124}<br>R_{64}<br>Y_{134}	A	$_{64}$-**R**FRVGK...AHLIG**R**SFNDAMSGQ**Y**-$_{134}$<br>αααααα...ααα-βββββ-------
2RGQ	R_{95}<br>R_{69}<br>Y_{89}	A<br>B	$_{69}$-**R**HCSSN...LTVVN**R**-$_{95}$<br>ββββββ...ββββ--<br>$_{89}$-**Y**LTVVN<br>βββββ-
1RI6	R_{50}<br>R_{66}<br>Y_{51}	A	$_{50}$-**RY**LYVGVRPEFRVLAY**R**-$_{66}$<br>-ββββββ----ββββββ
1RVK	R_{163}<br>R_{330}<br>Y_{339}	A	$_{163}$-**R**GYKGI...CRWYE**R**GLLHPFLE**Y**-$_{339}$<br>α---ββ...---ββββββ------
1RYL	R_{38}<br>R_{39}<br>Y_{6}	A<br>B	$_{38}$-**R**RLDID<br>-βββ--<br>$_{6}$-**Y**FAEID...LSGLR**R**-$_{39}$<br>ββββ--...αα---β
1S04	R_{31}<br>R_{25}<br>Y_{90}	A	$_{25}$-**R**LYDEK**R**RQIKP...VYRRF**Y**-$_{90}$<br>--αααααααα--...ααααα-

1T06	R_{225}<br>R_{223}<br>Y_{97}	B<br><br>A	$_{223}$-**R**G**R**-$_{225}$<br>---<br>$_{97}$-**Y**VVAVT<br>αααααα
1TT4	R_{177}<br>R_{289}<br>Y_{290}	A	$_{177}$-**R**FACAR...RFQAC**RY**-$_{290}$<br>---ααα...ααααααα
1UE8	R_{249}<br>R_{287}<br>Y_{2}	A	$_{2}$-**Y**DWFKQ...VEEAL**R**FSPPV...IASAN**R**-$_{287}$<br>αααααα...αααααααα---...ααααα-
1UF3	R_{218}<br>R_{6}<br>Y_{7}	A	$_{6}$-**RY**ILATS...SLLDL**R**-$_{218}$<br>--βββββ...ββββ--
**1UFA**	R_{86}<br>R_{83}<br>Y_{79}	A	$_{79}$-**Y**AKD**R**LE**R**-$_{86}$<br>αααααααα
1VBK	R_{50}<br>R_{7}<br>Y_{164}	A	$_{7}$-**R**YGEIG...FSRHG**R**IIVKT...GKAYI**Y**-$_{164}$<br>β-----...βββ--βββββ-...-βββββ
1VDW	R_{170}<br>R_{168}<br>Y_{90}	A<br><br>B	$_{168}$-**R**T**R**-$_{170}$<br>βββ<br>$_{90}$-**Y**NFING<br>αααααα
1VHK	R_{75}<br>R_{3}<br>Y_{144}	C	$_{3}$-**R**YFIEL...WTNEN**R**ELPIK...AAEQS**Y**-$_{144}$<br>βββ---...β----------...αααααα
1VI7	R_{104}<br>R_{53}<br>Y_{106}	A	$_{53}$-**R**HHCVA...TAVVV**R**Y**Y**-$_{106}$<br>----ββ...βββ-----
1VP4	R_{290}<br>R_{286}<br>Y_{65}	A	$_{65}$-**Y**TLQYS...PAITH**R**LAA**R**-$_{290}$ / α-helix<br>ααα---...-ααααααααα
1WDJ	R_{47}<br>R_{48}<br>Y_{148}	A	$_{47}$-**RR**SLQLA...VLVDP**Y**-$_{148}$<br>ααααααα...ββββ--
1WEK	R_{17}<br>R_{119}<br>Y_{120}	F	$_{17}$-**R**LFRIL...HALSL**RY**-$_{120}$<br>αααααα...ββββ---
**1XWM**	R_{188}<br>R_{192}<br>Y_{189}	A	$_{188}$-**RY**IE**R**-$_{192}$<br>ααααα
1Y7M	R_{159}<br>R_{159}<br>Y_{52}	A<br><br>B	$_{52}$-**Y**TIPYH...VPNGT**R**-$_{159}$<br>ααα-ββ...α----β<br>$_{159}$-**R**VTINR<br>βββββ-
1YW1	R_{345}<br>R_{360}<br>Y_{286}	A	$_{286}$-**Y**REFAE...EAKA**R**NLTLR...EM^{SE}AFP**R**-$_{345}$<br>αααααα...-------ααα...α α αα--
1YX1	R_{214}<br>R_{214}<br>Y_{211}	B<br><br>C	$_{211}$-**Y**WQ**R**-$_{214}$<br>αααα<br>$_{214}$-**R**LLQHF<br>αααα--
1YZ1	R_{21}<br>R_{152}<br>Y_{159}	B	$_{21}$-**R**EIADG...ALLDY**R**EDGVTP**Y**-$_{159}$<br>βββ---...βββββ-------β

2YZQ	R_{33}<br>R_{93}<br>Y_{91}	A	$_{33}$-**R**SFPVV...MELYD**Y**R**R**-$_{93}$<br>-βββββ...ααααα--β
2Z0J	R_{146}<br>R_{4}<br>Y_{15}	A<br><br>B	$_{146}$-**R**AGLDD<br>---ααα<br>$_{4}$-**R**VDVIPGEHLA**Y**-$_{15}$<br>βββ---------
1YEY	R_{100}<br>R_{151}<br>Y_{152}	A	$_{100}$-**R**WLGPE...DTIDF**RY**-$_{152}$<br>ααα---...ααα----
1ZL0	R_{264}<br>R_{57}<br>Y_{56}	B<br><br>A	$_{264}$-**R**IFGEY<br>αααααα<br>$_{56}$-**YR**-$_{57}$<br>--
1ZTV	R_{209}<br>R_{212}<br>Y_{78}	A<br><br>B	$_{209}$-**R**KK**R**-$_{212}$<br>αααα<br>$_{78}$-**Y**YASEE<br>α--ααα
**1ZYL**	R_{131}<br>R_{127}<br>Y_{128}	A	$_{127}$-**RY**LG**R**-$_{131}$<br>ααααα
3ILX	R_{61}<br>R_{125}<br>Y_{122}	A	$_{61}$-**R**VSSNT...RVITA**Y**PD**R**-$_{125}$<br>ββ----...ββββ-αααα
**2ACA**	R_{155}<br>R_{151}<br>Y_{152}	B	$_{151}$-**RY**RE**R**-$_{155}$<br>ααααα
3HN5	R_{173}<br>R_{153}<br>Y_{61}	A<br><br>B	$_{153}$-**R**VFFM^{SE}L...EHNVD**R**-$_{173}$<br>ββββ β β...----ββ<br>$_{61}$-**Y**AKHDP<br>αααα--
3HZE	R_{29}<br>R_{27}<br>Y_{31}	B<br><br>A	$_{29}$-**R**HYREQ<br>αααααα<br>$_{27}$-**R**QRH**Y**-$_{31}$<br>ααααα
1ZBO	R_{54}<br>R_{27}<br>Y_{28}	A	$_{27}$-**RY**LDM^{SE}VR...QGTEV**R**-$_{54}$<br>-ααα α αα...------
3IUK	R_{503}<br>R_{455}<br>Y_{514}	A	$_{455}$-**R**VVFDI...QFEFT**R**YLGWPGQAPS**Y**-$_{514}$<br>αααααα...αααααααααα-----αα
3K6C	R_{22}<br>R_{18}<br>Y_{6}	B	$_{6}$-**Y**FEPTQELSDET**R**DM^{SE}H**R**-$_{22}$<br>---αααα---αααα α αα
3KK4	R_{65}<br>R_{119}<br>Y_{64}	A	$_{64}$-**YR**GEAAN...NLYDS**Y**-$_{119}$<br>αα-----...ααα---
3K4W	R_{19}<br>R_{23}<br>Y_{87}	C<br><br>J	$_{19}$-**R**IYT**R**-$_{23}$<br>ααα--<br>$_{87}$-**Y**PDKFH<br>α---ββ
2QGQ	R_{389}<br>R_{224}<br>Y_{250}	A	$_{224}$-**R**VM^{SE}YLH...DKVVK**Y**FDVPV...KFLVG**R**-$_{389}$<br>ββ - ---...------βββ---...-βββββ

**3KH5**	R_{76}<br>R_{61}<br>Y_{67}	A	$_{67}$-**Y**NLI**R**EKHE**R**-$_{76}$<br>αααααααααα
**3LFZ**	R_{76}<br>R_{61}<br>Y_{67}	A	$_{67}$-**Y**NLI**R**EKHE**R**-$_{76}$<br>αααααααααα
3M6J	R_{19}<br>R_{8}<br>Y_{53}	D<br><br>A	$_{19}$-**R**WLSEI<br>αααααα<br>$_{8}$-**R**M^{SE}PLTV...WVREE**Y**-$_{53}$<br>- - -ααα...αααααα
3MEL	R_{129}<br>R_{126}<br>Y_{140}	C	$_{126}$-**R**QI**R**LCDRQNSIQY**Y**-$_{140}$<br>--βββββ--ββββββ
3NA2	R_{134}<br>R_{8}<br>Y_{122}	A<br><br>D	$_{134}$-**R**QLVFP<br>ααα---<br>$_{8}$-**R**IGQM^{SE}IL...AFTQR**Y**-$_{122}$<br>αααα α αα...αααααα
3NWZ	R_{59}<br>R_{67}<br>Y_{125}	B	$_{59}$-**R**EDGRFEV**R**LPIGP...PGM^{SE}GT**Y**-$_{125}$<br>------ββββ----...--- --β
3ONQ	R_{180}<br>R_{257}<br>Y_{179}	B	$_{179}$-**YR**NVYQV...AIGRM^{SE}**R**-$_{257}$<br>ααααααα...αααα α α
