# Supplementary material for: Rational Structure-Based Rescaffolding Approach to De Novo Design of Interleukin 10 (IL-10) Receptor-1 Mimetics
Source: PLoS One. 2016 Apr 28;11(4):e0154046. doi: 10.1371/journal.pone.0154046 (PMC4849758; doi:10.1371/journal.pone.0154046)
Supplement: S2 Table — M1: Ac-[KW2R3Y4D][KR7K8VD]R11A-NH2, M2: X-[KWR3Y4D][KR7K8VD]R11A-NH2 (X = 4-guanidinobutanoyl), M3: X-[KZR3Y4D][KR7K8VD]R11A-NH2 (X = 4-guanidinobutanoyl, Z = 5-Hydroxy-L-tryptophan), M4: Ac-R-1[KWR3Y4D][KR7K8VD]R11A-NH2, M5: Ac-E-2K-1[KWR3Y4D][KR7K8VD]R11A-NH2, M6: Ac-E-2R-1[KWR3Y4D][KR7K8VD]R11A-NH2. #H-bonds observed along MD simulations of 10 ns for IL-10/M1 and 30 ns for IL-10/M2-M6 are shown in black. Additional H-bonds observed during last 10 ns MD production for IL-10/M2-M6 are shown in gray. *Interactions via structural bridging water. S: side chain. M: main chain. aNot observed during the last 10 ns MD production. (PDF) [file pone.0154046.s008.pdf]

S2 Table. Summary of dynamic H-bond formation between IL-10 and IL-10R1 mimetics M1-M6 along MD simulations.[#]

**M1**	IL-10 interaction partner	**M2**	IL-10 interaction partner	**M3**	IL-10 interaction partner
		X (S)	IL-10$_{E50}$ (S)	X (S)	IL-10$_{L48}$ (M)<br>IL-10$_{L48}$* (M)<br>IL-10$_{E50}$ (S)<br>IL-10$_{E142}$*(S)<br>IL-10$_{Y149}$ (S)
W_2 (S)	IL-10$_{D144}$* (S, M)				
R_3 (S)	IL-10$_{D144}$ (S)	R_3 (S)	IL-10$_{D144}$ (S)	R_3 (S)	IL-10$_{E142}$ (S)[a]<br>IL-10$_{D144}$ (S)
Y_4 (S)	IL-10$_{K138}$ (S)<br>IL-10$_{E142}$ (S)	Y_4 (S)	IL-10$_{E142}$ (S)	Y_4 (S)	IL-10$_{K138}$ (S)<br>IL-10$_{E142}$ (S)
R_7 (S)	IL-10$_{Q38}$ (M)<br>IL-10$_{D41}$ (M)<br>IL-10$_{D41}$ (S)	R_7 (S)	IL-10$_{Q38}$ (M)<br>IL-10$_{D41}$ (S)<br>IL-10$_{L43}$ (M)<br>IL-10$_{D44}$ (S)	R_7 (S)	IL-10$_{D41}$ (S, M)<br>IL-10$_{Q38}$ (M)<br>IL-10$_{Q42}$ (M)[a]<br>IL-10$_{D44}$ (S)
K_8 (S)	IL-10$_{D44}$ (S)				
R_{11}(S)	IL-10$_{D44}$ (S)	R_{11}(S)	IL-10$_{D44}$ (S)<br>IL-10$_{Q42}$ (S)	R_{11}(S)	IL-10$_{D44}$ (S)

**M4**	IL-10 interaction partner	**M5**	IL-10 interaction partner	**M6**	IL-10 interaction partner
Ac	IL-10$_{D144}$*(S)	E_{-2} (S)<br><br><br>E_{-2} (M)	IL-10$_{K34}$ (S)<br>IL-10$_{Q38}$* (S)<br>IL-10$_{D144}$*(S)<br>IL-10$_{D144}$ (S)	E_{-2} (S)<br><br><br>E_{-2} (M)	IL-10$_{N148}$ (S)[a]<br>IL-10$_{K34}$ (S)<br>IL-10$_{D144}$ (S)<br>IL-10$_{D144}$*(S)
R_{-1} (S)	IL-10$_{L48}$ (M)<br>IL-10$_{L48}$* (M)<br>IL-10$_{E50}$ (S)<br>IL-10$_{E142}$*(S)<br>IL-10$_{Y149}$ (S)	K_{-1} (S)<br>K_{-1} (M)	IL-10$_{E50}$ (S)<br>IL-10$_{D144}$ (S)	R_{-1} (S)	IL-10$_{L48}$ (M)<br>IL-10$_{E50}$* (S)[a]<br>IL-10$_{E50}$ (S)<br>IL-10$_{Y149}$ (S)<br>IL-10$_{Y149}$*(S)[a]
R_3 (S)<br><br><br><br><br>R_3 (M)	IL-10$_{D41}$* (S)[a]<br>IL-10$_{E142}$ (S)<br>IL-10$_{S141}$* (S)[a]<br>IL-10$_{D144}$* (S)<br>IL-10$_{Q38}$* (S)<br>IL-10$_{Q38}$ (S)	R_3 (S)	IL-10$_{E142}$ (S)<br>IL-10$_{D144}$ (S)	R_3 (S)	IL-10$_{S141}$* (S)[a]<br>IL-10$_{E142}$ (S)<br>IL-10$_{D144}$ (S)<br>IL-10$_{D144}$* (S)
Y_4 (S)	IL-10$_{K138}$ (S)<br>IL-10$_{E142}$ (S)	Y_4 (S)	IL-10$_{D41}$* (S)[a]<br>IL-10$_{K138}$ (S)<br>IL-10$_{S141}$* (S)[a]<br>IL-10$_{E142}$ (S)	Y_4 (S)	IL-10$_{K138}$ (S)<br>IL-10$_{E142}$ (S)
R_7 (S)	IL-10$_{Q38}$ (M, S)<br>IL-10$_{D41}$ (S)<br>IL-10$_{L43}$ (M)<br>IL-10$_{D44}$ (S)	R_7 (S)	IL-10$_{Q38}$ (M)<br>IL-10$_{D41}$ (S, M[a])<br>IL-10$_{L43}$ (M)<br>IL-10$_{D44}$ (S)	R_7 (S)	IL-10$_{Q38}$ (M)<br>IL-10$_{D41}$ (S)<br>IL-10$_{L43}$ (M)<br>IL-10$_{D44}$ (S)
R_{11}(S)	IL-10$_{Q42}$ (S)[a]<br>IL-10$_{D44}$ (S)	R_{11}(S)	IL-10$_{Q42}$ (S)[a]<br>IL-10$_{D44}$ (S)<br>IL-10$_{D41*}$ (M)	R_{11}(S)	IL-10$_{D44}$ (S)<br>IL-10$_{D41*}$ (S)<br>IL-10$_{Q42}$ (S, M)

M1: Ac-[$KW_2R_3Y_4D$][KR_7K_8VD]$R_{11}A$-NH_2, **M2**: X-[KWR_3Y_4D][KR_7K_8VD]$R_{11}A$-NH_2 (X = 4-guanidinobutanoyl), **M3**: X-[KZR_3Y_4D][KR_7K_8VD]$R_{11}A$-NH_2 (X = 4-guanidinobutanoyl, Z = 5-Hydroxy-L-tryptophan), **M4**: Ac-R_{-1}[KWR_3Y_4D][KR_7K_8VD]$R_{11}A$-NH_2,
M5: Ac-$E_{-2}K_{-1}$[KWR_3Y_4D][KR_7K_8VD]$R_{11}A$-NH_2, **M6**: Ac-$E_{-2}R_{-1}$[KWR_3Y_4D][KR_7K_8VD]$R_{11}A$-NH_2.

[#]H-bonds observed along MD simulations of 10 ns for IL-10/**M1** and 30 ns for IL-10/**M2**-**M6** are shown in black. Additional H-bonds observed during last 10 ns MD production for IL-10/**M2-M6** are shown in gray. *Interactions via structural bridging water. S: side chain. M: main chain. [a]Not observed during the last 10 ns MD production.
