# Supplementary material for: Rational Structure-Based Rescaffolding Approach to De Novo Design of Interleukin 10 (IL-10) Receptor-1 Mimetics
Source: PLoS One. 2016 Apr 28;11(4):e0154046. doi: 10.1371/journal.pone.0154046 (PMC4849758; doi:10.1371/journal.pone.0154046)
Supplement: S3 Table — (PDF) [file pone.0154046.s009.pdf]

S3 Table. Binding free energies for alanine mutants of M1, M4 and M6 calculated with the MM-PBSA alanine scanning method.

IL10-R1 Mimetic mutants	Sequence	$\Delta G_{Mmutant\text{-}P}$ (kcal/mol)[a]	$\Delta\Delta G_{mutant}$ (kcal/mol)[b]	Residue Contribution (%)
**$M1_{R3A}$**	$Ac\text{-}[KWA_3Y_4D][KR_7K_8VD]R_{11}A\text{-}NH_2$	-22.6 ± 5.0	4.5 ± 8.3	17
**$M1_{Y4A}$**	$Ac\text{-}[KWR_3A_4D][KR_7K_8VD]R_{11}A\text{-}NH_2$	-22.4 ± 5.8	4.8 ± 8.8	18
**$M1_{R7A}$**	$Ac\text{-}[KWR_3Y_4D][KA_7K_8VD]R_{11}A\text{-}NH_2$	-17.5 ± 6.0	9.6 ± 9.0	35
**$M4_{R3A}$**	$Ac\text{-}R_{-1}[KWA_3Y_4D][KR_7K_8VD]R_{11}A\text{-}NH_2$	-49.8 ± 6.4	9.4 ± 9.5	16
**$M4_{Y4A}$**	$Ac\text{-}R_{-1}[KWR_3A_4D][KR_7K_8VD]R_{11}A\text{-}NH_2$	-49.0 ± 6.8	10.2 ± 9.8	17
**$M4_{R7A}$**	$Ac\text{-}R_{-1}[KWR_3Y_4D][KA_7K_8VD]R_{11}A\text{-}NH_2$	-28.7 ± 5.6	30.6 ± 8.9	52
**$M6_{R3A}$**	$Ac\text{-}E_{-2}R_{-1}[KWA_3Y_4D][KR_7K_8VD]R_{11}A\text{-}NH_2$	-59.2 ± 6.5	12.5 ± 10.3	17
**$M6_{Y4A}$**	$Ac\text{-}E_{-2}R_{-1}[KWR_3A_4D][KR_7K_8VD]R_{11}A\text{-}NH_2$	-60.0 ± 7.1	11.7 ± 10.7	16
**$M6_{R7A}$**	$Ac\text{-}E_{-2}R_{-1}[KWR_3Y_4D][KA_7K_8VD]R_{11}A\text{-}NH_2$	-41.4 ± 6.7	30.3 ± 10.4	42

[a] $\Delta G_{Mmutant\text{-}P}$ = Mimetic mutant-Protein binding free energy. [b] $\Delta\Delta G_{mutant}$ = change of MM-PBSA free energy of binding for alanine mimetic mutants in comparison to the non-mutated mimetics in complex with IL-10.
